# The actin remodeling protein cofilin is crucial for thymic αβ but not γδ T-cell development

**DOI:** 10.1371/journal.pbio.2005380

**Published:** 2018-07-09

**Authors:** Isabel Seeland, Ying Xiong, Christian Orlik, Daniel Deibel, Sandra Prokosch, Günter Küblbeck, Beate Jahraus, Daniela De Stefano, Sonja Moos, Florian C. Kurschus, Bernd Arnold, Yvonne Samstag

**Affiliations:** 1 Institute of Immunology, University of Heidelberg, Heidelberg, Germany; 2 Former Division of Molecular Immunology, German Cancer Research Center, Heidelberg, Germany; 3 Institute of Molecular Medicine, University Medical Center of the Johannes Gutenberg-University Mainz, Mainz, Germany; National Cancer Institute, United States of America

## Abstract

Cofilin is an essential actin remodeling protein promoting depolymerization and severing of actin filaments. To address the relevance of cofilin for the development and function of T cells in vivo, we generated knock-in mice in which T-cell–specific nonfunctional (nf) cofilin was expressed instead of wild-type (WT) cofilin. Nf cofilin mice lacked peripheral αβ T cells and showed a severe thymus atrophy. This was caused by an early developmental arrest of thymocytes at the double negative (DN) stage. Importantly, even though DN thymocytes expressed the TCRβ chain intracellularly, they completely lacked TCRβ surface expression. In contrast, nf cofilin mice possessed normal numbers of γδ T cells. Their functionality was confirmed in the γδ T-cell–driven, imiquimod (IMQ)-induced, psoriasis-like murine model. Overall, this study not only highlights the importance of cofilin for early αβ T-cell development but also shows for the first time that an actin-binding protein is differentially involved in αβ versus γδ T-cell development.

## Author summary

T cells are produced in the thymus and are critical to fighting infections and combating cancer. To move through the body and to fulfill their specific functions, T cells need to dynamically reshape their cell body. This requires remodeling the actin cytoskeleton using a plethora of actin-binding proteins, including cofilin. Whereas the majority of T cells use one type of cell membrane protein called αβ T cell receptor (TCR) to recognize their target, a minor population of T cells use a different type of receptors that are called γδTCR. The decision on whether thymocytes, the precursors of T cells, develop into αβTCR or γδTCR-bearing T cells takes place within the thymus. By replacing the cofilin gene with a nf copy, we identified an important role for cofilin in T-cell development. These mutant mice exhibited a severe thymus atrophy. Importantly, αβ T-cell development was severely affected, but the γδ T cells were unaffected in number and functional, as they were capable of responding to activation signal both in culture and inside the body. Overall, our study reveals the importance of cofilin in early αβ T-cell development and shows for the first time that an actin-binding protein is differentially involved in αβ versus γδ T-cell development.

## Introduction

One requirement for T-cell–mediated immune surveillance is the permanent reshaping of the cell body. Here, a functional remodeling of the actin cytoskeleton is important for changes of the cell shape during migration or immune synapse (IS) formation with antigen presenting cells (APCs) or target cells [1–5]. One protein that drives these actin dynamics is cofilin. Cofilin is a 19-kDa actin-binding protein that belongs to the actin depolymerizing factor (ADF)/cofilin family. In humans and mice, three different highly conserved isoforms are expressed [6,7]: nonmuscle cofilin (n-cofilin or cofilin-1 [Cfl1]) [8], muscle cofilin (m-cofilin or cofilin-2) [9], and destrin or ADF [10]. This study focused on Cfl1, which is highly expressed in T cells [11]. Cofilin has a dual function for actin dynamics, as it is both depolymerizing and severing actin filaments [12]. In resting human peripheral blood T cells (PBTs), cytoplasmic cofilin is constitutively phosphorylated at its serine 3 residue and thus inactive. Cofilin phosphorylation (inactivation) is mediated by LIM or testis-specific kinases (reviewed by Mizuno and colleagues [13]). Upon costimulation of resting T cells but not by TCR triggering alone, cofilin is dephosphorylated and thereby transmitted to its active state [11,14,15]. Once active, cofilin exerts its actin remodeling function which is crucial for proper IS formation and T-cell activation [16,17]. Dephosphorylated cofilin can also translocate to the nucleus where it may have anti-apoptotic functions and may enhance transcription [11,18]. It can furthermore serve as nuclear shuttle for actin [11,19], which is also involved in different nuclear mechanisms (reviewed by Falahzadeh and colleagues [20]). Besides T-cell costimulation, chemokine receptor triggering (e.g., by SDF-1α) can also lead to the dephosphorylation of cofilin [21]. In this regard, it was also shown that an active mitogen-activated protein kinase kinase (MEK) cofilin module is needed for T-cell movement [21], known to be driven by constant actin flow, i.e. migration in 3D environments [22–25]. The activity of cofilin is not only inhibited by phosphorylation but also by binding to phosphatidylinositol 4,5-bisphosphate (PIP_2_) near the plasma membrane and by a pro-oxidative microenvironment. Cofilin is inactivated by oxidation provoking T-cell hyporesponsiveness or in a long-term perspective necrotic-like programmed cell death [26,27]. In a reducing environment, however, even PIP_2_-bound cofilin becomes active, leading to enhanced actin dynamics in the vicinity of the plasma membrane [28].

Even though the essential role of cofilin for T-cell activation and migration was proven in in vitro studies of human PBTs, there is nothing known about the importance of cofilin for T-cell development in vivo. Thus, we created a mouse line in which T-cell–specific a nf form of cofilin was expressed instead of endogenous cofilin. The decision to use a cofilin knock-in rather than a knock-out mouse was due to the observation that knocking out a protein can result in an elevated expression of other proteins, which could in turn compensate for the lack of the protein of interest [29,30]. With the generated mice, we could show that cofilin is crucial for early αβ but not γδ T-cell development.

## Results

### Generation of a nf cofilin variant by addition of proline to the N-terminus

**Fig 1:**
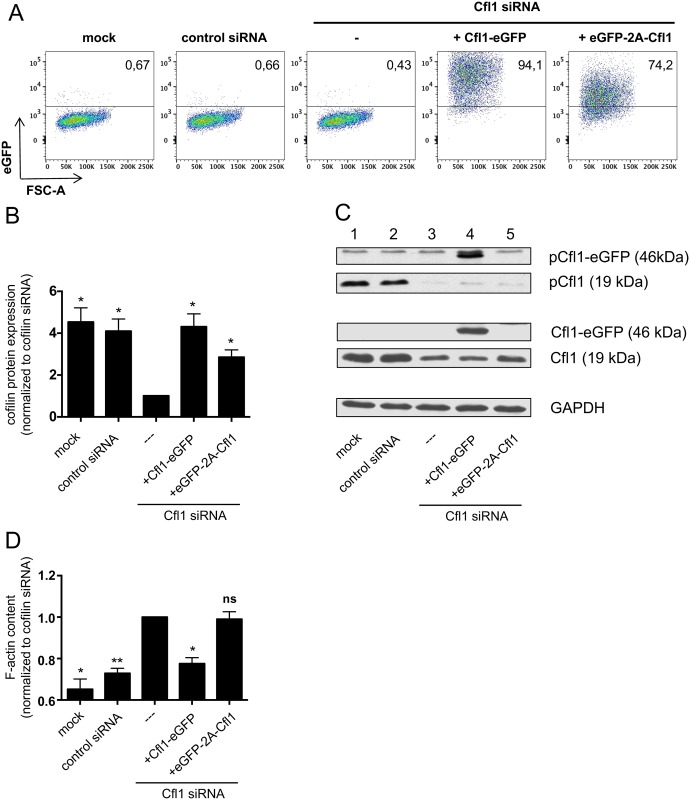
Cofilin expressed from an eGFP-2A-Cfl1 cassette is not functional in T-cells. Jurkat T cells were either transfected with no siRNA (mock), a nontargeting control siRNA or a cofilin-specific siRNA (binding to the 3′ UTR), in order to downregulate endogenous cofilin. Some of the cells that received cofilin siRNA were cotransfected with Cfl1-eGFP control vector or a vector carrying the eGFP-2A-Cfl1 sequence under the control of the CMV promoter. Cells were harvested and analyzed 48 h after transfection. (A) Exemplary flow cytometric analysis of eGFP expression of transfected Jurkat cells (*n* = 4 independent experiments). (B) Western blot analysis of total cell lysates by staining for total cofilin and GAPDH. All samples were normalized to cells transfected with cofilin siRNA, which was set as 1. Data is represented as mean ± SEM (*n* = 4 independent experiments). (C) Exemplary western blot showing pCfl1, total Cfl1, and control GAPDH staining. Endogenous cofilin has a size of 19 kDa (lanes 1–3 and 5), whereas cofilin derived from the Cfl1-eGFP control vector is expressed as eGFP fusion protein (size: 46 kDa, lane 4). (D) Total cellular F-actin content was analyzed by flow cytometric measurement of phalloidin binding. All samples were normalized to cells transfected with cofilin siRNA. Data is represented as mean ± SEM (*n* = 3 independent experiments). Significances were calculated against Jurkat cells transfected with siRNA only. ** *p* < 0.01; * *p* < 0.05. Underlying data can be found in S1 Data. Cfl1, cofilin-1; CMV, cytomegalovirus; GAPDH, glyceraldehyde-3-phosphate dehydrogenase; ns, not significant.

To overcome potential disadvantages of fusion proteins such as alterations of protein activity or subcellular localization, coexpression of fluorescent dyes together with the protein of interest is widely used to monitor protein expression and/or promoter activities. With the help of the viral 2A consensus motif, two proteins can be coexpressed from a single mRNA by a mechanism called “ribosome skipping” [31–33]. Upon cotranslational cleavage, most of the 2A sequence remains attached to the C-terminus of the upstream protein, whereas only a single proline stays attached to the N-terminus of the downstream protein. Cofilin is reported to undergo cotranslational processing at its N-terminus and its activity is post-translationally regulated by (de)phosphorylation at its serine 3 residue. We wondered whether or not addition of proline to cofilin’s N-terminus would lead to its inactivation. Therefore, we created a plasmid in which an enhanced green fluorescent protein (eGFP)-2A-Cfl1 expression cassette was cloned under a cytomegalovirus (CMV) promoter. To test expression and functionality of cofilin derived from the eGFP-2A-Cfl1 expression cassette, the plasmid was transfected into Jurkat leukemia cells in which the endogenous cofilin was knocked down via siRNA. A vector in which the C-terminus of cofilin was fused to eGFP served as positive control. Transfection efficiency and successful expression of the eGFP-2A-Cfl1 cassette was visible by eGFP analysis (Fig 1A). Cofilin protein expression was further confirmed by western blot analysis of total Jurkat cell lysates (Fig 1B).

We first examined whether the addition of proline to cofilin’s N-terminus influences cofilin phosphorylation. Comparing phosphorylation of endogenous cofilin (Fig 1C, lane 1, pCfl1) and the cofilin from the eGFP-2A-Cfl1 cassette (Fig 1C, lane 5, pCfl1) revealed much less phosphorylation of the latter. Note that cofilin expressed from the positive control vector (Fig 1C, lane 4, pCfl1-eGFP) showed no alteration in the phosphorylation state.

To test the functionality of nonphosphorylated cofilin encoded by eGFP-2A-Cfl1, the F-actin content of transfected Jurkat cells was determined by analysis of phalloidin binding via flow cytometry (Fig 1D). As expected, Jurkat cells with a successful cofilin knock-down showed an increase in total F-actin. Cotransfection with a positive control vector, which expressed eGFP-tagged WT cofilin, rescued F-actin depolymerization. However, Jurkat cells with cofilin knock-down that expressed eGFP-2A-Cfl1 harbored a similar high F-actin content as cells transfected with siRNA only. Thus, even though cofilin from the eGFP-2A-Cfl1 plasmid was expressed and less phosphorylated, it was not functional pointing towards defective regulation by phosphorylation.

Overall, cofilin expressed from the eGFP-2A-Cfl1 vector showed a defect in both phosphorylatability and actin remodeling function.

### Generation of T-cell–specific nf cofilin knock-in mice

Having observed the functional inactivity of cofilin obtained from the eGFP-2A-Cfl1 expression cassette in vitro, we wondered about the consequences of cofilin dysfunction in T cells in vivo. Therefore, we generated mice expressing an eGFP-2A-Cfl1 expression cassette instead of endogenous cofilin specifically in T cells. Thus, the nf form of cofilin should be expressed only in T cells. The targeting strategy used for generation of knock-in mice is shown in S1A Fig. In short, an eGFP-2A-Cfl1 expression cassette was inserted into the intronic region between exon 1 and 2 of the mouse cofilin gene. To prevent transcription of the cassette, a floxed stop cassette was included in front. Another locus of X (cross)-over in P1 (loxP) site was cloned into the noncoding region of exon 1. T-cell–specific knock-out of endogenous cofilin and knock-in of the eGFP-2A-Cfl1 expression cassette was achieved by crossing mice carrying the construct with lymphocyte-specific protein-tyrosine kinase (Lck)-Cre mice that express Cre recombinase under the proximal p56^lck^ (Lck) promoter [34]. WT mice (Cfl1^+/+^) could be discriminated from heterozygous (Cfl1^+/nf^) and homozygous knock-in mice (Cfl1^nf/nf^) by PCR (S1B Fig). All mice were born with an expected Mendelian ratio and developed without apparent signs of abnormality. Rarely, Cfl1^nf/nf^ mice showed inflamed cheeks or intestinal prolapses. Successful T-cell–specific knock-in of the expression cassette was confirmed by flow cytometry (via eGFP expression; S1C Fig). Please note that eGFP positive cells were already detected in heterozygous DN1 thymocytes (S1D Fig) but not in common lymphoid progenitors in the bone marrow. This is also in line with earlier studies investigating the activity of the Lck proximal promoter [35].

### Characterization of the nf cofilin mutant

To further characterize the nf cofilin mutant, cofilin obtained from T cells of Cfl1^+/nf^ mice (expressing both WT and nf cofilin) and control B6 mice was subjected to mass spectrometry (S1E Fig). Besides its post-translational regulation by phosphorylation, cofilin was reported to undergo N-terminal excision of the initiator methionine followed by acetylation of alanine (Uniprot; P18760). Accordingly, mass spectrometry analysis of cofilin from B6 T cells revealed the presence of peptides starting with acetylated alanine (S1E Fig). Thereby, peptides were either phosphorylated at serine 3 (Ac+Ph) or dephosphorylated (Ac) (S1E Fig, left). These two peptide species (Ac and Ac+Ph) were also identified in MS/MS analysis of cofilin obtained from T cells of Cfl1^+/nf^ mice, which express both wt and nf cofilin (S1E Fig, right). Additionally, a N-terminal cofilin peptide starting with proline, followed by methionine and alanine was found only in Cfl1^+/nf^ mice. In this peptide, no serine phosphorylation and, due to the N-terminal proline-methionine, also no alanine acetylation could be detected. Thus, in the generated knock-in mice, the single remaining proline residue hinders co- and post-translational processing of cofilin in T cells.

### Loss of cofilin function leads to a massive decrease of peripheral T-cells

**Fig 2:**
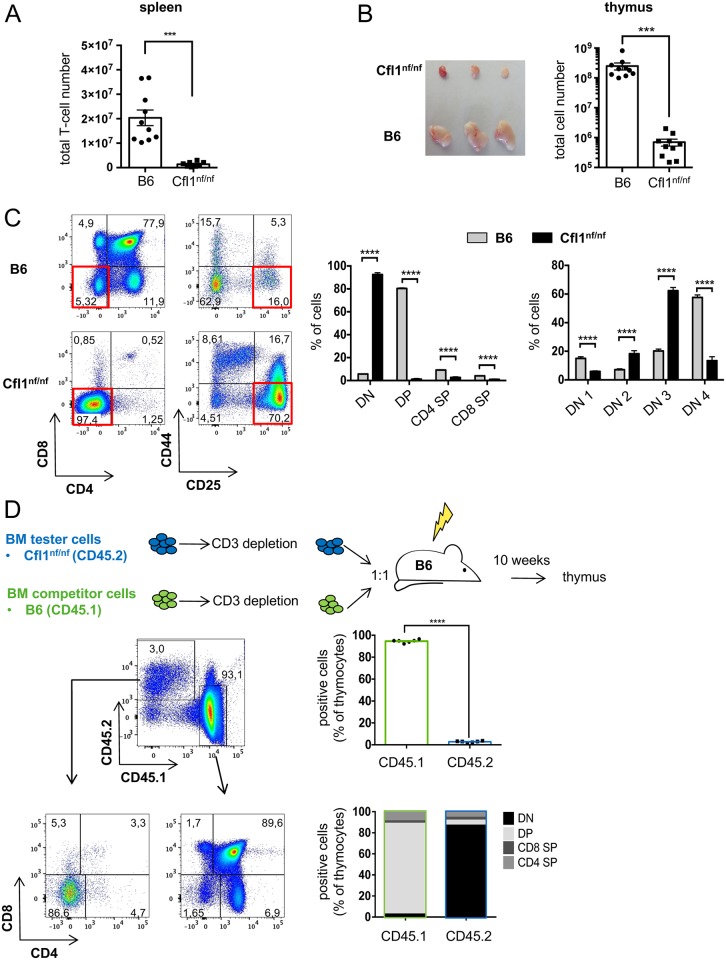
Mice expressing nf cofilin show a severe thymus atrophy and a developmental arrest at the DN3 stage. (A) Total T-cell number in spleen of B6 mice and nf cofilin knock-in mice (*n* = 6 independent experiments with a total of ≥8 mice per group). (B) Thymus was isolated and weighed, and the total cell number was determined from 4–5-weeks-old B6 or nf cofilin knock-in mice (*n* = 6 independent experiments with a total of ≥10 mice per group). (C) Flow cytometric analysis of thymocyte differentiation by CD4, CD8, CD25, and CD44 staining (*n* ≥ 8 mice per group). Exemplary dot blots from representative mice are shown on the left, whereas the statistical evaluation of summary data is shown in the middle (for DN, DP, and SP stages) and on the right (for DN cell stages). (D) Creation of mixed bone marrow chimera. Lethally irradiated B6 mice were reconstituted with equal numbers of CD3^+^ cell–depleted BM cells from CD45.2^+^ tester (Cfl1^nf/nf^) and CD45.1^+^ competitor (B6) mice. Total chimerism was measured and CD4 versus CD8 plots show the developmental stage of thymocytes derived from CD45.1^+^ or CD45.2^+^ BM cells. Plots are representative of six mixed chimeras per group. Bar graphs show the average abundance of each major thymocyte population within the chimera from both tester (CD45.2^+^) and competitor (CD45.1^+^) donor cells. Data is represented as mean ± SEM. **** = p<0.0001; ** = p<0.01. Underlying Data can be found in S1 Data. BM, bone marrow; DN, double negative; DP, double positive; SP, single positive.

Having established homozygous mice expressing nf cofilin in T-cells, we next characterized their immune cells. Nf cofilin knock-in mice (Cfl1^nf/nf^) had similar numbers of total splenocytes as wt B6 animals (S2A Fig). However, their lymph node (LN) cell numbers were significantly diminished (S2B Fig). Further analysis of leukocyte cell populations revealed that Cfl1^nf/nf^ mice show a massive decrease in T-cell populations both in percentage and numbers in spleen (Fig 2A, S2A Fig). The almost complete lack of T cells in the periphery was accompanied by an absolute increase in other leukocyte cell populations such as splenic B-cells, natural killer (NK) cells, and dendritic cells (DCs) as well as eosinophils and neutrophils (S2C Fig). This finding explains why total cell numbers in the spleen were normal despite the nearly complete loss of T cells in Cfl1^nf/nf^ mice. Note that mice carrying the nf cofilin construct homozygously without Cre-mediated knock-in and also mice carrying the nf cofilin construct heterozygously with Cre-mediated knock-in had similar T-cell populations as B6 mice (S2D Fig).

### Cofilin is essential for early T-cell development in the thymus

In regard to the small T-cell numbers in the periphery, we next investigated the thymic development of nf cofilin knock-in mice. Here, Cfl1^nf/nf^ mice showed a severe thymus atrophy, which was characterized by a decrease in the thymic cell number of more than 99% (Fig 2B). Flow cytometric characterization demonstrated that thymocytes were almost exclusively found within the CD4^-^ CD8^-^ double negative (DN) stage, suggesting a very early block in T-cell development (Fig 2C, left bar chart). The DN stage can be further discriminated into 4 developmental steps by differential expression of surface CD25 and CD44 [36]. Within the DN stage, thymocytes were mainly detected in the DN2 (CD44^+^ CD25^+^) and DN3 (CD44^-^ CD25^+^) stage, with most cells accumulating at the DN3 stage. Furthermore, a loss of cells in the DN4 stage (CD44^-^ CD25^-^) was observed (Fig 2C, right bar chart).

T-cell development is not determined solely by T-cell progenitors themselves but is also influenced by the thymic stroma. To test whether the reason for impaired thymocyte development is T-cell intrinsic, mixed bone marrow chimeras were created (Fig 2D). To this end, irradiated B6 mice were injected with a 1:1 ratio of bone marrow (BM) tester cells (derived from Cfl1^nf/nf^ knock-in mice; CD45.2^+^) and control competitor cells (B6; CD45.1^+^). Once successful reconstitution was verified in peripheral blood of the recipient mice, their thymus was taken out and cells were analyzed by flow cytometry. Thymocytes that originated from BM of Cfl1^nf/nf^ accumulated in the DN stage (mainly in DN3), whereas control competitor cells derived from B6 mice developed completely normally. B-cells that originated from BM of Cfl1^nf/nf^ mice developed to a normal extent (S2E Fig). This indicates that the disturbed T-cell development in nf cofilin knock-in mice is caused by T-cell intrinsic factors. Further, the number of CD45.2^+^ cells which were found in the thymi of reconstituted B6 mice was much smaller than the one of CD45.1^+^ control cells (3% versus 95%), implying not only a developmental but also a proliferative disadvantage of cells which originated from BM of homozygous knock-in mice. Overall, the severe thymus atrophy seems to be caused by a lack of DN thymocyte expansion.

### Importance of cofilin for αβ but not γδ T-cell development

**Fig 3:**
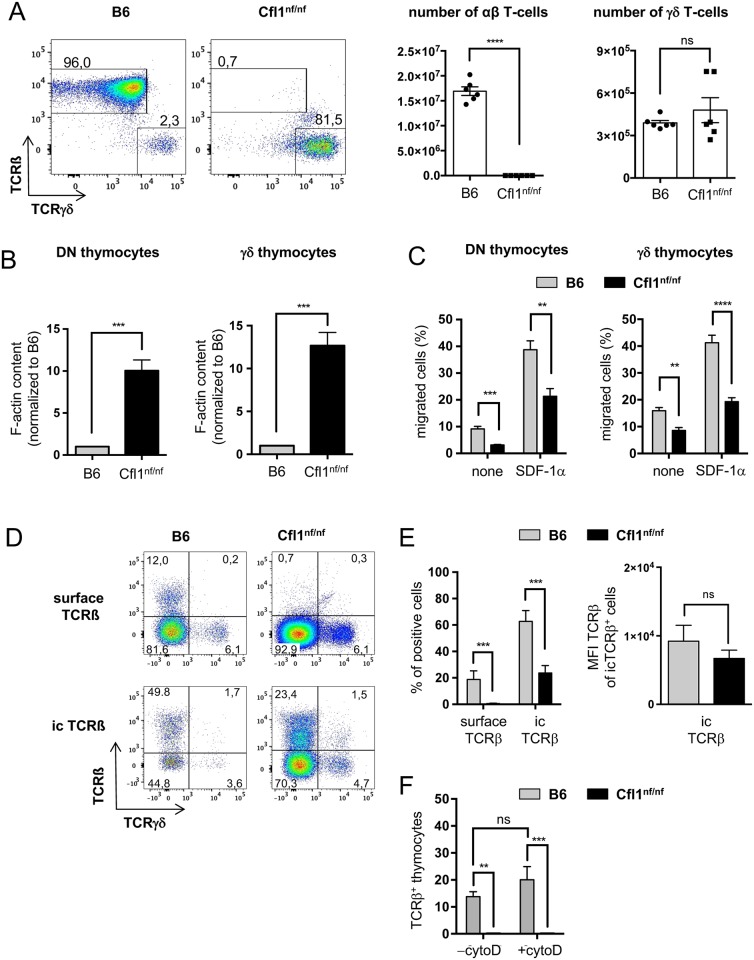
DN thymocytes of Cfl1^nf/nf^ mice show a dramatically enhanced F-actin content and impaired migratory capacity as well as a lack of TCRβ surface expression. (A) CD3^+^ splenocytes were analyzed for expression of TCRβ and TCRγδ. Shown are representative dot blots (left panels) and calculated absolute cell numbers (right panel) of TCRβ and TCRγδ expressing cells. Data is represented as mean ± SEM and summarizes 3 independent experiments with a total of 6 mice per group. (B) Total F-actin amount of DN thymocytes or γδ thymocytes was determined by SiR-actin staining (*n* = 3 independent experiments with a total ≥6 mice per group). (C) Migratory capacity of DN cells or γδ thymocytes was determined in a transwell assay (pore size 5 μm) in which SDF-1α (200 ng/ml) was used as chemotactic stimulus. Migration was carried out for 3 h (*n* = 3 independent experiments with ≥4 mice per group). (D) TCRβ surface (surface TCRβ) and intracellular (ic TCRβ) expression was analyzed in DN cells by flow cytometry. Representative dot plots from TCRβ versus TCRγδ staining on B6 and Cfl1^nf/nf^ DN cells are shown (*n* = 4 independent experiments with a total of ≥7 mice per group). (E) Analysis of surface and ic expression of TCRβ in DN cells of B6 (grey bar) and Cfl1^nf/nf^ mice (black bar) (left bar chart). Analysis of MFI of TCRβ of icTCRβ^+^ DN cells of B6 (grey bar) and Cfl1^nf/nf^ mice (black bar) (right bar chart). (F) Analysis of surface expression of TCRβ in DN thymocytes of B6 and Cfl1^nf/nf^ mice before (-cytoD) and after cytochalasin D treatment (+cytoD). Data is represented as mean ± SEM. **** *p* < 0.0001; *** *p* < 0.001; ** *p* < 0.01; * *p* < 0.05; Underlying data can be found in S1 Data. cytoD, cytochalasin D; ic, intracellular; MFI, mean fluorescence intensity nf, nonfunctional; ns, not significant.

Despite the enormous thymus atrophy and reduction in peripheral T-cell numbers, there were few CD3^+^ cells detected in secondary lymphoid organs of Cfl1^nf/nf^ mice. Hence, we wondered if the remaining peripheral T-cells are of a specific subtype. Analysis of CD4 and CD8 expression in T-cells from the spleen revealed a strong accumulation of CD4^-^ CD8^-^ cells in nf cofilin knock-in mice (S3A Fig). We next checked splenic T-cells for TCRβ and TCRγδ surface expression. In B6 mice >95% of T-cells are of the αβ subtype and only a minor fraction of γδ T-cells are found (~ 2%) (Fig 3A). In contrast, Cfl1^nf/nf^ mice do not harbor substantial amounts of αβ T-cells but possess normal absolute numbers of γδ T-cells (Fig 3A, bars on the right). Note that also the distinct CD4^-^CD8^+^ population of splenic T-cells isolated from Cfl1^nf/nf^ mice expressed exclusively TCRγδ but not TCRβ (S3B Fig).

γδ T cells’ survival is not due to a missing Lck-Cre mediated knock-in of nf cofilin, as γδ T-cells do express Lck [37]. Moreover, successful recombination of the nf cofilin construct was confirmed by PCR and the expression of the cofilin protein in γδ T-cells was confirmed by flow cytometry (S3C and S3D Fig). Thus, cofilin appears to be essential only for αβ but not γδ T-cells.

### In early thymocytes, lack of cofilin function leads to an accumulation of F-actin, a defective migration, and impaired TCRß but normal TCRγδ surface expression

As shown above, mice with a T-cell–specific knock-in of nf cofilin almost completely lacked peripheral αβ T-cells and showed a severe thymus atrophy. Residual thymocytes, which were found, accumulated in the DN stage. Thus, we next addressed why thymocytes arrested particularly at this stage of T-cell development and why expression of nf cofilin is critical for αβ but not γδ T-cell development. In regard to the actin depolymerizing function of cofilin, we first checked the cellular F-actin content of DN thymocytes as well as of thymic γδ T-cells. The F-actin content in DN thymocytes was highly increased in cells obtained from Cfl1^nf/nf^ mice in comparison to those derived from B6 mice (Fig 3B, left bar chart). Interestingly, also thymic γδ T cells from Cfl1^nf/nf^ mice accumulated more F-actin than control γδ T cells (Fig 3B, right bar chart).

Besides cofilin, destrin is another actin depolymerizing factor that can be expressed in mammalian cells. Flow cytometric studies revealed that destrin is expressed in DN and γδ thymocytes and its expression is not impaired in Cfl1^nf/nf^ mice (S4A Fig). Thus, the presence of destrin could not compensate the effects of nf cofilin.

One important process during early T-cell development, which may be influenced by altered actin dynamics, is the outward migration of DN thymocytes from the entry site at the corticomedullary junction (CMJ) to the outer cortex. To determine the migratory capacity of DN thymocytes and γδ T cells that express nf cofilin, we employed a transwell assay, in which SDF-1α, the natural ligand of CXCR4, was used as chemotactic stimulus. DN thymocytes from Cfl1^nf/nf^ mice showed both a decreased random migration (Fig 3C, left bar chart; none) and a diminished directed migration (Fig 3C, left bar chart; +SDF-1α). A similar reduction in the migratory potential was observed for thymic γδ T cells (Fig 3C, right bar chart).

Note that the decreased migratory capacity of nf cofilin–expressing cells was not due to a lack of CXCR4, which was expressed intracellularly and extracellularly to a similar extent as in control cells (S4B Fig).

A second process during T-cell development, which requires actin dynamics, is the redistribution of receptors to the cell surface, as e.g. the TCR. While normal surface expression of TCRγδ was observed in DN thymocytes of Cfl1^nf/nf^ mice, they completely lacked TCRβ surface expression (Fig 3D, upper panel). However, TCRβ was detected inside nf cofilin expressing DN thymocytes (Fig 3D, lower panel). Although the number of icTCRβ^+^ DN thymocytes was decreased in Cfl1^nf/nf^ compared to B6 mice, the mean fluorescence intensity (MFI) of TCRβ in icTCR^+^ cells was similar between Cfl1^nf/nf^ and B6 mice (Fig 3E).

To test whether the surface expression of TCRβ in Cfl1^nf/nf^ mice can be rescued by the disruption of actin filaments (e.g., cortical actin), we treated DN thymocytes with cytochalasin D (cytoD). Although TCRβ surface expression on thymocytes of B6 mice was slightly but not significantly enhanced after cytoD treatment, thymocytes of Cfl1^nf/nf^ mice showed still no TCRβ on their surface (Fig 3F).

Our data demonstrate that DN thymocytes as well as thymic γδ T-cells from Cfl1^nf/nf^ mice showed a strong accumulation of F-actin and a decreased migration capacity. However, only TCRβ but not TCRγδ surface expression was abolished in thymocytes of nf cofilin knock-in mice.

### Expression of nf cofilin does not interfere with the development of different subsets of γδ T cells

**Fig 4:**
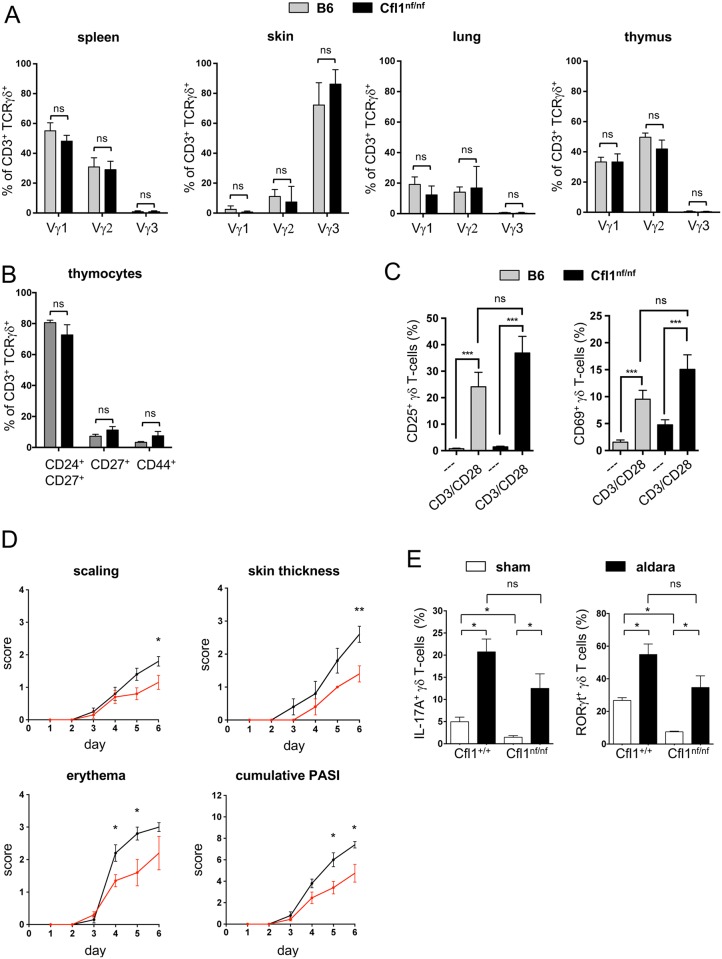
Cfl1^nf/nf^ mice show normal γδ T-cell subsets which remain functional. (A) Analysis of the surface expression of Vγ1, Vγ2, and Vγ3 of γδ T-cells isolated from the spleen, skin, lung, and thymus of B6 (grey bar) or Cfl1^nf/nf^ mice (black bar) (*n* = 4 independent experiments with ≥4 mice per group). (B) Analysis of the surface expression of CD24, CD27, and CD44 of γδ thymocytes of B6 (grey bar) or Cfl1^nf/nf^ mice (black bar) (*n* = 4 independent experiments with ≥4 mice per group). (C) In vitro activation of splenic γδ T cells of Cfl1^nf/nf^ (black bar) and control mice (grey bar) by plate-bound CD3 and CD28 antibodies for 24 h. Determination of the T-cell activation markers CD25 (left bar chart) and CD69 (right bar chart) by flow cytometry. (D) Age- and sex-matched Cfl1^nf/nf^ (red line) and Cfl1^+/+^ (black line) mice at 7 weeks of age were used for an IMQ-induced psoriasis-like model. Over 6 days, prior to topical application of IMQ, scores of individual parameters such as scaling, back skin thickness, and erythema formation were measured and the accumulated PASI was calculated. (E) Flow cytometric analysis of IL-17A and RORγt expression in γδ T cells of skin-draining LNs. Cytokine production was assessed after 6 days of topical application of IMQ containing Aldara crème (Sham) or control crème (Aldara) (experiment with ≥4 mice per group). Data are represented as mean ± SEM. **** *p* < 0.0001; *** *p* < 0.001; ** *p* < 0.01; * *p* < 0.05; Underlying data can be found in S1 Data. Cfl1, cofilin-1; IMQ, imiquimod; ns, not significant; PASI, psoriasis area severity index.

Since the heterogeneous γδ T-cell compartment consists of different subpopulations, the influence of nf cofilin on specific γδ T-cell populations was tested. First, the surface expression of Vγ1, Vγ2, and Vγ3 chains was analyzed on thymic or peripheral (skin, lung, spleen) γδ T cells (Fig 4A). As expected, these Vγ chains were tissue specifically expressed. However, comparing the tissue specific Vγ chain expression of γδ T-cells from B6 and Cfl1^nf/nf^ revealed no differences at all. In line with these results, distinct γδ T-cell populations, which are characterized by the expression of different surface markers on γδ thymocytes (CD24, CD27, and CD44), were similar in Cfl1^nf/nf^ and B6 mice (Fig 4B).

### γδ T cells of Cfl1^nf/nf^ mice remain functionally active

Since knock-in mice had no αβ T cells but normal numbers of γδ T cells in the periphery, we wondered whether γδ T cells are still functional. To evaluate their functionality, purified splenic γδ T cells were in vitro stimulated by anti-CD3 and anti-CD28 antibodies for 24 h. Stimulated γδ T cells of both control and Cfl1^nf/nf^ mice showed increased expression of T-cell activation markers (CD25, CD69) compared to unstimulated cells (Fig 4C).

To further investigate the functionality of γδ T cells in Cfl1^nf/nf^ mice under in vivo conditions, we chose the IMQ-induced psoriasis-like murine model. In this, the loss of γδ T cells was already linked to diminished psoriasis-like symptoms [38]. By applying IMQ containing Aldara crème topically on the shaved back skin of either WT (Cfl^+/+^) or Cfl1^nf/nf^ knock-in mice for 6 days, the psoriasis-like phenotype was assessed (Fig 4D). After 3 days, both groups started to show signs of scaling, skin thickening, and erythema. Cfl1^nf/nf^ mice exhibited slightly decreased erythema at day 4 and day 5 and diminished scaling and skin thickening at day 6 compared to WT mice. Also, the cumulative psoriasis area severity index (PASI) score was partially reduced in Cfl1^nf/nf^ mice at day 5 and 6. Nevertheless, Cfl1^nf/nf^ mice developed strong psoriasis-like symptoms over the course of the experiment and also the severity of inflammation increased up to the end of the experiment.

IL-17A–producing and RORγt-positive γδ T cells are crucial for proper development of psoriasis [39,40]. Therefore, we tested the IL-17A production and RORγt expression in skin-draining LN γδ T cells. Ex vivo restimulation of LN cells from control vehicle crème (Sham) or IMQ-treated mice revealed a slightly but nonsignificantly reduced percentage of IL-17A, producing as well as RORγt-expressing γδ T cells in Cfl1^nf/nf^ mice compared to WT mice. Nonetheless, both groups showed significantly increased percentages of these cells after IMQ application compared to the mice treated with control crème only (Fig 4E). Together, these experiments demonstrated that γδ T cells of Cfl1^nf/nf^ mice are functional and able to induce a psoriasis-like skin inflammation in the absence of αβ T cells.

## Discussion

Using knock-in mice in which T-cell-specific nf cofilin was expressed instead of endogenous cofilin, we demonstrate that cofilin is essential for αβ but not γδ T-cell development. Cfl1^nf/nf^ mice lacked peripheral αβ T cells and showed a severe thymus atrophy, which was caused by an early developmental arrest at the DN stage. DN thymocytes exhibited impaired actin dynamics, a defective migratory capacity, and a lack of TCRβ surface expression (Fig 3). Even though γδ thymocytes were also impaired in actin dynamics and cell motility, nf cofilin knock-in mice harbored normal γδ T-cell numbers in the periphery. Those γδ T cells showed normal expression of Vγ chains and also the different subpopulations (discriminated via CD24, CD27, and CD44) were similar to those of γδ T cells from B6 mice. Thus, nf cofilin does not interfere with the development of different subsets of γδ T cells.

The functionality of peripheral γδ T cells from nf cofilin knock-in mice was proven in in vitro and in vivo experiments. First, in vitro CD3/CD28 stimulation confirmed the ability of splenic γδ T cells to be activated, although nf cofilin is expressed instead of WT cofilin. Second, we analyzed these mice via the γδ T cell–driven, IMQ-induced psoriasis-like murine model. Even though the cumulative PASI score was decreased in IMQ-treated Cfl1^nf/nf^ mice compared to control mice, they developed clear psoriasis-like symptoms, and also, the severity of psoriasis-like skin inflammation increased within the course of the experiment. In line with this, nf cofilin knock-in mice exhibited a strong induction of IL-17A^+^ RORγt^+^ γδ T cells in skin-draining LNs of IMQ-treated animals.

In this study, we avoided knocking out cofilin completely and rather expressed a nf cofilin mutant. This was accomplished solely by the addition of a single proline to cofilin’s N-terminus by making use of the viral 2A sequence. The additional proline inhibits removal of the initiator methionine and as a consequence also N-terminal alanine acetylation. Moreover, no phosphorylation on serine 3 was detected. In the nf cofilin mutant, the lack of cofilin phosphorylation is most likely due to impaired cotranslational processing, which could render cofilin less accessible for kinases. Thereby, less phosphorylation does not automatically mean more activity. Similar findings were obtained with oxidized cofilin, which is a poor target for LIM kinase and was found to be less phosphorylated than untreated cofilin even though it is not able to remodel the actin cytoskeleton [27].

One consequence of the expression of nf cofilin was the drastically increased F-actin content of DN thymocytes. Functionally, a massive accumulation of actin filaments could cause a stiffening of the respective cells and could render them less dynamic. Indeed, early thymocytes of nf cofilin knock-in mice showed a decreased migratory capacity towards SDF-1α. Also, their spontaneous undirected migration in the absence of chemokines was diminished. In the postnatal thymus, DN1 cells are mainly found in the inner cortex (close to the CMJ) before they start an outward migration during their transition to DN2 and DN3 stage [41]. Thymocytes that are not able to migrate outward from the CMJ to the cortex due to deficiency of CXCR4, the chemokine receptor of SDF-1α, are not developing beyond the DN stage [42]. Another independent study of thymocytes derived from CXCR4-deficient progenitor cells also revealed that their development is already drastically altered before they develop into DP thymocytes [43]. Thus, the diminished migratory capacity of early thymocytes of nf cofilin knock-in mice—possibly due to stiffening of the actin cytoskeleton—may at least in part play a role for their developmental arrest. Moreover, in human PBTs, cofilin was shown to be dephosphorylated and thereby activated in lamellipodia upon triggering of cells with SDF-1α [21]. Thus, it is likely that also during thymocyte development, cofilin is one of the effector molecules, which get activated by chemokines secreted by thymic epithelial cells and are involved in the directed migration of DN cells to the outer cortex. Although normal numbers of γδ T cells were found in nf cofilin knock-in mice, γδ thymocytes also showed an accumulation of F-actin as well as a defective migratory capacity as observed for DN thymocytes. These findings imply that cofilin function and high cell motility are more crucial for αβ than for γδ T-cell development.

Besides the essential role of actin dynamics for cell movement, the dynamic rearrangement of the actin cytoskeleton is also important for clustering and (re)distribution of surface receptors, e.g., during immune synapse formation [44–46]. In our study, knock-in of a dysfunctional cofilin had detrimental effects on TCRβ but not on TCRγδ surface expression of DN thymocytes. Even though the TCRβ chain is rearranged and expressed intracellularly, it is not detectable on the the cell surface. Interestingly, the surface translocation of other proteins, e.g. CD25, CD44, or CXCR4, was not influenced, indicating a selective effect of nf cofilin on TCRβ surface translocation rather than an interference with the general surface transport of membrane proteins.

Alternatively to altered TCRβ transport to the cell surface, the diminished TCRβ expression among DN thymocytes could theoretically also be due to the lack of NKT cells, since the Lck promoter is also active in these cells [35]. However, NKT cells were still present in nf cofilin mice. Interestingly, these NK1.1^+^ DN thymocytes did also not express TCRβ on their surface, emphasizing the importance of cofilin for TCRβ translocation.

TCRβ surface expression is essential for pre-TCR signaling and the transition through the so-called β-checkpoint. Interestingly, thymocytes of nf cofilin knock-in mice accumulated in the DN3 stage, a phenotype which is indeed characteristic for impaired pre-TCR signaling (e.g., caused by knocking out components of the pre-TCR [47,48]). Whereas functional pre-TCR signaling and passage through the β-checkpoint induces extensive proliferation of DN thymocytes and development into DP thymocytes, those cells which are not able to express a functional pre-TCR get eradicated by apoptosis [49,50]. This proliferative burst is one of the key functions of pre-TCR signaling, and thus the dramatic thymic atrophy in nf cofilin knock-in mice is at least in part due to the lack of proliferation and/or induction of apoptosis. However, to finally conclude that nf cofilin is interfering with preTCR signaling, a comparison between TCRa^-/-^ and Cfl1^nf/nf^ mice would be necessary.

In the thymus, *Tcrb*, *Tcrg*, and *Tcrd* are all rearranged at the DN2/3 stage of development. It is at the DN3 stage in which final fate determination of αβ and γδ lineages takes place. If cells have rearranged the TCRβ chain, the β-selection process starts. In contrast to the αβ lineage cells, those DN3 cells that have rearranged functional γ and δ chains undergo γδ selection remain negative for both T-cell co-receptors and develop into γδ T cells (for details about the αβ versus γδ lineage decision, see the publication of Zarin and colleagues [51]). As αβ and γδ T cells undergo different developmental processes, they may also have varying requirements, e.g., in regard to up-regulation of receptors. This study shows that cofilin-driven cellular processes are essential for cell surface expression of TCRβ but appear to be less important for γδ TCR up-regulation. One possible explanation for this could be that other actin-remodeling proteins can partially compensate for the lack of cofilin function in γδ T cells. We investigated destrin, another closely related actin-depolymerizing factor. It was equally expressed in DN and γδ thymocytes of Cfl1^nf/nf^ mice. This shows first that destrin could not compensate the effects of nf cofilin (massive increase of the F-actin amount in nf cofilin knock-in cells) and second that normal developmental and function of γδ thymocytes was not due to a higher expression of destrin. So far, we have no information about the expression and function of other actin-depolymerizing proteins. Furthermore, we did not find any information in the literature about the role of actin remodeling proteins in γδ T-cells. Additionally, there is no other study—at least to our knowledge—reporting about a differential role of an actin remodeling protein for αβ versus γδ T-cell development.

Previous studies in which cytoskeletal proteins were targeted in mice revealed that they are of major importance for the emigration of mature SP thymocytes from the thymus to secondary lymphoid organs. However, these proteins play only a minor role for early thymocyte development. This holds true for mice deficient in mDia (actin-nucleating-polymerizing protein) [52] as well as for L-plastin (actin bundling protein) [53] and Coronin1A (inhibits nucleation-promoting Arp2/3 complex) [54,55]. Closest to the phenotype observed for nf cofilin knock-in mice—albeit being more modest—was the phenotype of mice with a knock-out of WASP (Wiskott–Aldrich Syndrome protein). These mice exhibited a reduction of thymic cellularity and a relative increase in DN3 cells among the DN cell compartment [56]. However, in another study in which WASP was targeted, there was no effect on thymocyte development [57]. A study of Zhang and colleagues, in which T cells expressed WASP with a deleted VCA domain on the WASP knock-out background confirmed the importance of WASP for T-cell development [58]. DN cells from those mice do express pre-TCRα and TCRβ. However, in contrast to nf cofilin knock-in mice, they develop DP cells. Yet DP cells also do not express TCRβ on the cell surface and show a surface phenotype resembling the one of immature thymocytes from the DN population. Thus, even though WASP seems to be important for pre-TCR signaling and thymocyte development, it most likely plays only a partial role in this process, as there are still thymocytes which develop beyond the DN stage in WASP knock-out mice, and also, mature T-cells are present in their periphery.

Together, our data demonstrate the unique role of cofilin activity for proper development of αβ but not γδ T cells. Probably, cofilin and related signaling cascades are valuable starting points to decipher differences in developmental checkpoints for αβ versus γδ T-cell lineage decision. Besides this, usage of the Cre/lox system also allows us to knock-in the functionally inactive cofilin in other cell types. Our strategy not only allows the expression of a nf form of cofilin but also the coexpression of eGFP, which further enables to monitor knock-in cells and cofilin promoter activity. This makes the generated mice to a valuable tool for studying the relevance of cofilin in different cell types.

## Methods

### Ethics statement

All mouse experiments were carried out in accordance with the relevant guidelines and regulations by the federal state Baden-Wuerttemberg and Rhineland-Palatinate, Germany. Psoriasis experiments were approved by Landesuntersuchungsamt Rheinland-Pfalz (TVA # G13-1-099). To dissect lymphoid organs (e.g., LNs, spleen, or thymus), mice were euthanized by cervical dislocation or lethal dose of CO_2_.

### Mice

Strain details as well as procedure to generate nf cofilin knock-in mice are provided in S1 Text. All mice were bred and maintained at the central animal facility of the University of Heidelberg under specific pathogen-free conditions. Mice used in experiments were sex- and age-matched and were generally 6–12 weeks (or, for thymic experiments, 4–5 weeks) old.

### Transfection of Jurkat cells

For knock-down of endogenous cofilin in Jurkat cells and expression of eGFP-2A-Cfl1, Jurkat cells were transfected with the Bio-Rad GenePulser II. To this end, each 10 Mio of cells were mixed with 2 μg cofilin siRNA (CFL1 ON-TARGETplus siRNA; Dharmacon) and/or 15 μg plasmid DNA and electroporated at 230 V and 950 mF. Cells were cultured in RPMI1640 medium containing 10% FCS at 37 °C and 5% CO_2_. Transfection efficacy as well as successful down-modulation of endogenous cofilin was examined by western blot (see S1 Text).

### F-actin content

For determination of the cellular F-actin content of Jurkat cells, 1 Mio of cells were fixed with 1.5% PFA in PBS for 10 min at 37 °C. Afterwards, cells were permeabilized in FACS buffer (PBS with 0.5% BSA) containing 0.1% saponine for 10 min at RT. Cells were stained in the same buffer containing Phalloidin-AF647 (Life technologies) for 20 min at RT. For determination of the F-actin content of DN thymocytes or γδ thymocytes, cells were stained with 500 nM SiR-actin (Cytoskeleton, Inc.) for 3 h at 37 °C. The higher the MFI of Phalloidin-AF647 or SiR-actin, the more filamentous actin is present inside the cell.

### Migration assay

Chemotaxis of thymocytes was tested with 5-μm pore size Transwell plates (Corning). To this end, 50,000 cells in medium were plated in the upper compartment of the transwell insert and medium +/− 200 ng/ml SDF-1α (Peprotech) was added into the lower compartment. Migration was carried out for 3 h at 37 °C. The number of transmigrated thymocytes was determined via flow cytometry by making use of an internal bead standard (BD Biosciences).

### CytoD treatment

To disrupt the cortical actin of thymocytes, 2 Mio cells were treated with 20 μM cytochalasin D (Sigma Aldrich) for 1 h at 37 °C. After cytoD treatment, cells were stained for TCRβ and surface expression was analyzed by flow cytometry.

### In vitro T-cell stimulation

Splenic γδ T cells were MACS isolated using “TCRg/d T-cell isolation kit, mouse” (Miltenyi Biotec). Cells numbering 300,000 were stimulated by plate-bound αnti-CD3 (10 μg/mL, BD Bioscience) and anti-CD28 antibodies (2 μg/mL, BD Bioscience) for 24 h at 37 °C. After stimulation, cells were stained for surface markers and analyzed by flow cytometry.

### In vivo IMQ-induced psoriasis-like model

Age- and sex-matched Cfl1^nf/nf^ and Cfl1^+/+^ mice at 7 weeks of age were used for IMQ-induced psoriasis-like model.

Mice received a daily topical dose of 50 mg of IMQ crème (5%) (Aldara, Meda Pharma) or 50 mg of a control vehicle crème (Sham crème, University medicine Mainz) over 6 days. Prior to topical application scores of individual parameters such as back skin thickness, scaling, and erythema formation were measured and the accumulated PASI was calculated as described previously [59]. At day 6, mice were killed humanely and LN cells were isolated for intracellular cytokine staining.

### PMA/Ionomycin ex vivo stimulation of isolated LNs

To induce cytokine production, single cell suspensions of isolated LNs were stimulated with 50 ng/ml phorbol 12-myristate 13-acetate (PMA, Sigma Aldrich) and 500 ng/ml ionomycin (Sigma Aldrich) in the presence of 1 μg/ml Brefeldin A for 4 h at 37 °C. After stimulation, cells were stained for surface markers and intracellular cytokines and analyzed by flow cytometry.

### Statistical analysis

Statistical analysis was performed with Prism 6 software. Values are expressed as mean ± SEM. Unpaired two-tailed Student *t* test was used to test significant numerical differences between groups. Differences of *p* ≤ 0.05 were considered to be statistically significant (* *p* ≤ 0.05; ** *p* ≤ 0.01; *** *p* ≤ 0.001; **** *p* < 0.0001).

## Supporting information

S1 Fig Generation of T-cell–specific nf cofilin knock-in mice. (A) Strategy used to create T-cell–specific nf cofilin knock-in mice. The first line shows the exon-intron organization of the mouse cofilin gene. It lies on chromosome 19 and consists of 4 exons (filled yellow boxes). In the targeted allele (second line) a floxed stop cassette, an eGFP-2A-Cfl1 sequence and a FRT-flanked neomycin (neo) cassette were inserted into the intronic region between exon 1 and 2 of the cofilin gene. Another loxP site was introduced into the noncoding sequence of exon 1. Mice carrying the construct in their germline were mated with Flp deleter mice in order to remove the neomycin cassette (third line). Afterwards, T-cell–specific knock-out of endogenous cofilin by deletion of exon 1 and at the same time knock-in of the eGFP-2A-Cfl1 expression cassette was achieved by crossing mice carrying the Flp recombined construct with Lck-Cre mice. (B) Mouse genotyping was performed by PCR of tail DNA. The allele carrying the construct could be discriminated from the WT allele by the additional loxP site. Cfl1^+/+^: wt mice; Cfl1^+/nf^: heterozygous mice; Cfl1^nf/nf^: homozygous mice. (C) Flow cytometric analysis of eGFP expression in T cells and non-T cells of purified peripheral blood mononuclear cells PBMCs from Cfl1^+/nf^ mice. (D) Flow cytometric analysis of eGFP expression in common lymphoid progenitor cells CLPs from the bone marrow and thymocytes (DN1, DP and SP stage) from thymi of Cfl1^+/nf^ mice. For analysis of eGFP expression in CLPs, lineage negative cells were isolated from BM of mice by MACS. CLPs were then identified by their expression of IL7Rα, c-kit and Sca-1 [60]. (E) LC-MS/MS analysis of cofilin peptides resulting from tryptic digestion of cofilin isolated from splenic T cells of B6 and Cfl1^+/nf^ mice. Shown are the extracted ion chromatograms of the depicted peptides. “Ac” represents N-terminus of cofilin starts with acetylated alanine and serine is not phosphorylated; “Ac + Ph” represents N-terminus of cofilin starts with acetylated alanine and serine is phosphorylated; “PMAS” represents N-terminus of cofilin starts with proline, followed by methionine, alanine and non-phosphorylated serine. CLP, common lymphoid progenitor cells; PBMC, peripheral blood mononuclear cell; WT, wild-type.
(TIF)

S2 Fig T-cell–specific expression of nf cofilin leads to a massive reduction of peripheral T cells. (A) Total spleen cell number and percentage of T cells in spleen of B6 mice and Cfl1^nf/nf^ mice. (B) Total thymic cell number and percentage of T cells in LNs of B6 mice and Cfl1^nf/nf^ mice. (C) Splenic cells were analyzed for B-cell, NK cell, DC, neutrophil, and eosinophil populations. Shown are the percentage of total splenocytes. Each data point represents an individual mouse. (D) Flow cytometric analysis of B- and T-cell populations in lymphocytes derived from LNs of control B6 mice, Cfl1^+/+^ mice (homozygous for construct but no Cre-mediated knock-in), Cfl1^nf/wt^ (heterozygous for construct with Cre-mediated knock-in) and Cfl1^nf/nf^ mice (homozygous for construct with Cre-mediated knock-in). One representative result out of 3 independent experiments with a total of 6 mice per group is shown. (E) Analysis of the percentage of splenic B-cells within the chimera (see Fig 2D) from both tester (CD45.2^+^) and competitor (CD45.1^+^) donor cells. Data is represented as mean ± SEM and summarizes 4 independent experiments with a total of ≥ 6 mice per group. **** *p* < 0.0001; ** *p* < 0.01; * *p* < 0.05. Underlying data can be found in S1 Data. ns, not significant.
(TIF)

S3 Fig Remaining peripheral T cells are of γδ T-cell subset type expressing nf cofilin. (A) Flow cytometric analysis of T-cell co-receptors CD4 and CD8 on splenic T cells of B6 and Cfl1^nf/nf^ mice. (B) Flow cytometric analysis of T-cell populations in lymphocytes derived from spleen of control B6 mice (left panel) and Cfl1^nf/nf^ mice (right panel). CD8^+^ T-cell population in spleen of B6 mice express either highly TCRβ or low amounts of TCRγδ. Splenic CD8^+^ T cells of Cfl1^nf/nf^ mice express solely TCRγδ. (C) γδ T cells were isolated from splenocytes of Cfl1^nf/nf^ mice via FACS sort and were analyzed for Cre recombination by PCR of cell lysates. Lysates of thymocytes were used as a positive control, whereas mouse tail DNA (from Cfl1^nf/nf^ mice) and H_2_O served as negative controls. (D) Cofilin expression analysis of splenic γδ T cells of B6 mice (upper panel) and Cfl1^nf/nf^ mice (lower panel). Cells were pre-gated on CD3^+^ γδ T cells. nf, nonfunctional.
(TIF)

S4 Fig Cfl1^nf/nf^ mice show normal destrin as well as CXCR4 expression. (A) Analysis of destrin expression in DN and γδ thymocytes of B6 and Cfl1^nf/nf^ mice. (B) Analysis of ic and surface expression of CXCR4. Data is represented as mean ± SEM and summarizes 4 independent experiments with a total of ≥ 6 mice per group. **** *p* < 0.0001; ** *p* < 0.01; * *p* < 0.05. Underlying data can be found in S1 Data. ic, intracellular; ns, not significant.
(TIF)

S1 Text Supplemental experimental procedures. (DOCX)

S1 Data Underlying data. Data for Figs 1B, 1D, 2A–2D, 3A–3C, 3E, 3F and 4A–4E, S2A–S2C, S2E, S4A and S4B Figs.
(XLSX)

## Abbreviations

ADF: actin depolymerizing factor
APC: antigen presenting cell
BM: bone marrow
Cfl1: cofilin-1
CLP: common lymphoid progenitor cells
CMJ: corticomedullary junction
CMV: cytomegalovirus
cytoD: cytochalasin
DC: dendritic
DN: double negative
DP: double positive
eGFP: enhanced green fluorescent protein
GAPDH: glyceraldehyde-3-phosphate dehydrogenase
ic: intracellular
IMQ: imiquimod
IS: immune synapse
Lck: lymphocyte-specific protein-tyrosine kinase
LN: lymph node
loxP: locus of X(cross)-over in P1
MEK: mitogen-activated protein kinase kinase
MFI: mean fluorescence intensity
nf: nonfunctional
NK: natural killer
ns: not significant
PASI: psoriasis area severity index
PBMC: peripheral blood mononuclear cell
PBT: peripheral blood T cell
PIP_2_: phosphatidylinositol 4,5-bisphosphate
SP: single positive
TCR: T cell receptor
WASP: Wiskott–Aldrich Syndrome protein
WT: wild-type

## References

[1] Burkhardt JK, Carrizosa E, Shaffer MH. The actin cytoskeleton in T cell activation. Annu Rev Immunol. 2008;26:233–59. 10.1146/annurev.immunol.26.021607.090347 18304005

[2] Angus KL, Griffiths GM. Cell polarisation and the immunological synapse. Curr Opin Cell Biol. 2013;25(1):85–91. 10.1016/j.ceb.2012.08.013 22990072 PMC3712171

[3] Piragyte I, Jun CD. Actin engine in immunological synapse. Immune Netw. 2012;12(3):71–83. 10.4110/in.2012.12.3.71 22916042 PMC3422712

[4] Samstag Y, Eibert SM, Klemke M, Wabnitz GH. Actin cytoskeletal dynamics in T lymphocyte activation and migration. J Leukoc Biol. 2003;73(1):30–48. 12525560 10.1189/jlb.0602272

[5] Kumari S, Curado S, Mayya V, Dustin ML. T cell antigen receptor activation and actin cytoskeleton remodeling. Biochim Biophys Acta. 2014;1838(2):546–56. 10.1016/j.bbamem.2013.05.004 23680625 PMC3877165

[6] Bernstein BW, Bamburg JR. ADF/cofilin: a functional node in cell biology. Trends Cell Biol. 2010;20(4):187–95. 10.1016/j.tcb.2010.01.001 20133134 PMC2849908

[7] Vartiainen MK, Mustonen T, Mattila PK, Ojala PJ, Thesleff I, Partanen J, et al The three mouse actin-depolymerizing factor/cofilins evolved to fulfill cell-type-specific requirements for actin dynamics. Mol Biol Cell. 2002;13(1):183–94. 10.1091/mbc.01-07-0331 11809832 PMC65081

[8] Nishida E, Maekawa S, Sakai H. Cofilin, a protein in porcine brain that binds to actin filaments and inhibits their interactions with myosin and tropomyosin. Biochemistry. 1984;23(22):5307–13. 6509022 10.1021/bi00317a032

[9] Abe H, Ohshima S, Obinata T. A cofilin-like protein is involved in the regulation of actin assembly in developing skeletal muscle. J Biochem. 1989;106(4):696–702. 2691511 10.1093/oxfordjournals.jbchem.a122919

[10] Moriyama K, Nishida E, Yonezawa N, Sakai H, Matsumoto S, Iida K, et al Destrin, a mammalian actin-depolymerizing protein, is closely related to cofilin. Cloning and expression of porcine brain destrin cDNA. J Biol Chem. 1990;265(10):5768–73. 2156828

[11] Samstag Y, Eckerskorn C, Wesselborg S, Henning S, Wallich R, Meuer SC. Costimulatory signals for human T-cell activation induce nuclear translocation of pp19/cofilin. Proc Natl Acad Sci U S A. 1994;91(10):4494–8. 8183936 10.1073/pnas.91.10.4494 PMC43812

[12] Condeelis J. How is actin polymerization nucleated in vivo? Trends Cell Biol. 2001;11(7):288–93. 11413039 10.1016/s0962-8924(01)02008-6

[13] Mizuno K. Signaling mechanisms and functional roles of cofilin phosphorylation and dephosphorylation. Cell Signal. 2013;25(2):457–69. 10.1016/j.cellsig.2012.11.001 23153585

[14] Samstag Y, Bader A, Meuer SC. A serine phosphatase is involved in CD2-mediated activation of human T lymphocytes and natural killer cells. J Immunol. 1991;147(3):788–94. 1677669

[15] Samstag Y, Henning SW, Bader A, Meuer SC. Dephosphorylation of pp19: a common second signal for human T cell activation mediated through different accessory molecules. Int Immunol. 1992;4(11):1255–62. 1472477 10.1093/intimm/4.11.1255

[16] Eibert SM, Lee KH, Pipkorn R, Sester U, Wabnitz GH, Giese T, et al Cofilin peptide homologs interfere with immunological synapse formation and T cell activation. Proc Natl Acad Sci U S A. 2004;101(7):1957–62. 10.1073/pnas.0308282100 14762171 PMC357034

[17] Lee KH, Meuer SC, Samstag Y. Cofilin: a missing link between T cell co-stimulation and rearrangement of the actin cytoskeleton. Eur J Immunol. 2000;30(3):892–9. 10.1002/1521-4141(200003)30:3<892::AID-IMMU892>3.0.CO;2-U 10741406

[18] Samstag Y, John I, Wabnitz GH. Cofilin: a redox sensitive mediator of actin dynamics during T-cell activation and migration. Immunol Rev. 2013;256(1):30–47. 10.1111/imr.12115 24117811 PMC3884758

[19] Pendleton A, Pope B, Weeds A, Koffer A. Latrunculin B or ATP depletion induces cofilin-dependent translocation of actin into nuclei of mast cells. J Biol Chem. 2003;278(16):14394–400. 10.1074/jbc.M206393200 12566455

[20] Falahzadeh K, Banaei-Esfahani A, Shahhoseini M. The potential roles of actin in the nucleus. Cell J. 2015;17(1):7–14. 10.22074/cellj.2015.507 25870830 PMC4393673

[21] Klemke M, Kramer E, Konstandin MH, Wabnitz GH, Samstag Y. An MEK-cofilin signalling module controls migration of human T cells in 3D but not 2D environments. EMBO J. 2010;29(17):2915–29. 10.1038/emboj.2010.153 20676060 PMC2944044

[22] Friedl P, Entschladen F, Conrad C, Niggemann B, Zanker KS. CD4+ T lymphocytes migrating in three-dimensional collagen lattices lack focal adhesions and utilize beta1 integrin-independent strategies for polarization, interaction with collagen fibers and locomotion. Eur J Immunol. 1998;28(8):2331–43. 10.1002/(SICI)1521-4141(199808)28:08<2331::AID-IMMU2331>3.0.CO;2-C 9710211

[23] Wolf K, Muller R, Borgmann S, Brocker EB, Friedl P. Amoeboid shape change and contact guidance: T-lymphocyte crawling through fibrillar collagen is independent of matrix remodeling by MMPs and other proteases. Blood. 2003;102(9):3262–9. 10.1182/blood-2002-12-3791 12855577

[24] Woolf E, Grigorova I, Sagiv A, Grabovsky V, Feigelson SW, Shulman Z, et al Lymph node chemokines promote sustained T lymphocyte motility without triggering stable integrin adhesiveness in the absence of shear forces. Nat Immunol. 2007;8(10):1076–85. 10.1038/ni1499 17721537

[25] Lammermann T, Bader BL, Monkley SJ, Worbs T, Wedlich-Soldner R, Hirsch K, et al Rapid leukocyte migration by integrin-independent flowing and squeezing. Nature. 2008;453(7191):51–5. 10.1038/nature06887 18451854

[26] Wabnitz GH, Goursot C, Jahraus B, Kirchgessner H, Hellwig A, Klemke M, et al Mitochondrial translocation of oxidized cofilin induces caspase-independent necrotic-like programmed cell death of T cells. Cell Death Dis. 2010;1:e58 10.1038/cddis.2010.36 21364663 PMC3032559

[27] Klemke M, Wabnitz GH, Funke F, Funk B, Kirchgessner H, Samstag Y. Oxidation of cofilin mediates T cell hyporesponsiveness under oxidative stress conditions. Immunity. 2008;29(3):404–13. 10.1016/j.immuni.2008.06.016 18771940

[28] Schulte B, John I, Simon B, Brockmann C, Oelmeier SA, Jahraus B, et al A reducing milieu renders cofilin insensitive to phosphatidylinositol 4,5-bisphosphate (PIP2) inhibition. J Biol Chem. 2013;288(41):29430–9. 10.1074/jbc.M113.479766 24003227 PMC3795243

[29] De Souza AT, Dai X, Spencer AG, Reppen T, Menzie A, Roesch PL, et al Transcriptional and phenotypic comparisons of Ppara knockout and siRNA knockdown mice. Nucleic Acids Res. 2006;34(16):4486–94. 10.1093/nar/gkl609 16945951 PMC1636368

[30] Rossi A, Kontarakis Z, Gerri C, Nolte H, Holper S, Kruger M, et al Genetic compensation induced by deleterious mutations but not gene knockdowns. Nature. 2015;524(7564):230–3. 10.1038/nature14580 26168398

[31] Ryan MD, King AM, Thomas GP. Cleavage of foot-and-mouth disease virus polyprotein is mediated by residues located within a 19 amino acid sequence. Journal of General Virology. 1991;72 (Pt 11):2727–32. 1658199 10.1099/0022-1317-72-11-2727

[32] Ryan MD, Donnelly M, Lewis A, Mehrotra AP, Wilkie J, Gani D. A model for nonstoichiometric, cotranslational protein scission in eukaryotic ribosomes. Bioorganic Chemistry. 1999;27(1):55–79.

[33] Donnelly MLL, Luke G, Mehrotra A, Li XJ, Hughes LE, Gani D, et al Analysis of the aphthovirus 2A/2B polyprotein 'cleavage' mechanism indicates not a proteolytic reaction, but a novel translational effect: a putative ribosomal 'skip'. Journal of General Virology. 2001;82:1013–25. 10.1099/0022-1317-82-5-1013 11297676

[34] Orban PC, Chui D, Marth JD. Tissue- and site-specific DNA recombination in transgenic mice. Proc Natl Acad Sci U S A. 1992;89(15):6861–5. 1495975 10.1073/pnas.89.15.6861 PMC49604

[35] Shimizu C, Kawamoto H, Yamashita M, Kimura M, Kondou E, Kaneko Y, et al Progression of T cell lineage restriction in the earliest subpopulation of murine adult thymus visualized by the expression of lck proximal promoter activity. Int Immunol. 2001;13(1):105–17. 11133839 10.1093/intimm/13.1.105

[36] Godfrey DI, Kennedy J, Suda T, Zlotnik A. A developmental pathway involving four phenotypically and functionally distinct subsets of CD3-CD4-CD8- triple-negative adult mouse thymocytes defined by CD44 and CD25 expression. J Immunol. 1993;150(10):4244–52. 8387091

[37] Laird RM, Hayes SM. Roles of the Src tyrosine kinases Lck and Fyn in regulating gammadeltaTCR signal strength. PLoS One. 2010;5(1):e8899 10.1371/journal.pone.0008899 20126650 PMC2811189

[38] Cai Y, Shen X, Ding C, Qi C, Li K, Li X, et al Pivotal role of dermal IL-17-producing gammadelta T cells in skin inflammation. Immunity. 2011;35(4):596–610. 10.1016/j.immuni.2011.08.001 21982596 PMC3205267

[39] Becher B, Pantelyushin S. Hiding under the skin: Interleukin-17-producing gammadelta T cells go under the skin? Nat Med. 2012;18(12):1748–50. 10.1038/nm.3016 23223063

[40] Pantelyushin S, Haak S, Ingold B, Kulig P, Heppner FL, Navarini AA, et al Rorgammat+ innate lymphocytes and gammadelta T cells initiate psoriasiform plaque formation in mice. J Clin Invest. 2012;122(6):2252–6. 10.1172/JCI61862 22546855 PMC3366412

[41] Lind EF, Prockop SE, Porritt HE, Petrie HT. Mapping precursor movement through the postnatal thymus reveals specific microenvironments supporting defined stages of early lymphoid development. J Exp Med. 2001;194(2):127–34. 11457887 10.1084/jem.194.2.127 PMC2193450

[42] Plotkin J, Prockop SE, Lepique A, Petrie HT. Critical role for CXCR4 signaling in progenitor localization and T cell differentiation in the postnatal thymus. J Immunol. 2003;171(9):4521–7. 14568925 10.4049/jimmunol.171.9.4521

[43] Ara T, Itoi M, Kawabata K, Egawa T, Tokoyoda K, Sugiyama T, et al A role of CXC chemokine ligand 12/stromal cell-derived factor-1/pre-B cell growth stimulating factor and its receptor CXCR4 in fetal and adult T cell development in vivo. J Immunol. 2003;170(9):4649–55. 12707343 10.4049/jimmunol.170.9.4649

[44] Billadeau DD, Nolz JC, Gomez TS. Regulation of T-cell activation by the cytoskeleton. Nat Rev Immunol. 2007;7(2):131–43. 10.1038/nri2021 17259969

[45] Ritter AT, Angus KL, Griffiths GM. The role of the cytoskeleton at the immunological synapse. Immunol Rev. 2013;256(1):107–17. 10.1111/imr.12117 24117816 PMC4312978

[46] Thauland TJ, Hu KH, Bruce MA, Butte MJ. Cytoskeletal adaptivity regulates T cell receptor signaling. Sci Signal. 2017;10(469). 10.1126/scisignal.aah3737 PMC5854469 28270556

[47] Fehling HJ, Krotkova A, Saint-Ruf C, von Boehmer H. Crucial role of the pre-T-cell receptor alpha gene in development of alpha beta but not gamma delta T cells. Nature. 1995;375(6534):795–8. 10.1038/375795a0 7596413

[48] Malissen M, Gillet A, Ardouin L, Bouvier G, Trucy J, Ferrier P, et al Altered T cell development in mice with a targeted mutation of the CD3-epsilon gene. EMBO J. 1995;14(19):4641–53. 7588594 10.1002/j.1460-2075.1995.tb00146.x PMC394561

[49] vonBoehmer H. Control of T-cell development by the Pre-T and alpha beta T-cell. Receptor Activation by Antigens, Cytokines, Hormones, and Growth Factors. 1995;766:52–61. 10.1111/j.1749-6632.1995.tb26648.x 7486699

[50] Aifantis I, Mandal M, Sawai K, Ferrando A, Vilimas T. Regulation of T-cell progenitor survival and cell-cycle entry by the pre-T-cell receptor. Immunol Rev. 2006;209:159–69. 10.1111/j.0105-2896.2006.00343.x 16448541

[51] Zarin P, Chen EL, In TS, Anderson MK, Zuniga-Pflucker JC. Gamma delta T-cell differentiation and effector function programming, TCR signal strength, when and how much? Cell Immunol. 2015;296(1):70–5. 10.1016/j.cellimm.2015.03.007 25866401

[52] Sakata D, Taniguchi H, Yasuda S, Adachi-Morishima A, Hamazaki Y, Nakayama R, et al Impaired T lymphocyte trafficking in mice deficient in an actin-nucleating protein, mDia1. J Exp Med. 2007;204(9):2031–8. 10.1084/jem.20062647 17682067 PMC2118705

[53] Morley SC, Wang C, Lo WL, Lio CW, Zinselmeyer BH, Miller MJ, et al The actin-bundling protein L-plastin dissociates CCR7 proximal signaling from CCR7-induced motility. J Immunol. 2010;184(7):3628–38. 10.4049/jimmunol.0903851 20194718 PMC2855624

[54] Foger N, Rangell L, Danilenko DM, Chan AC. Requirement for coronin 1 in T lymphocyte trafficking and cellular homeostasis. Science. 2006;313(5788):839–42. 10.1126/science.1130563 16902139

[55] Shiow LR, Roadcap DW, Paris K, Watson SR, Grigorova IL, Lebet T, et al The actin regulator coronin 1A is mutant in a thymic egress-deficient mouse strain and in a patient with severe combined immunodeficiency. Nat Immunol. 2008;9(11):1307–15. 10.1038/ni.1662 18836449 PMC2672406

[56] Zhang J, Shehabeldin A, da Cruz LAG, Butler J, Somani AK, McGavin M, et al Antigen receptor-induced activation and cytoskeletal rearrangement are impaired in Wiskott-Aldrich syndrome protein-deficient lymphocytes. Journal of Experimental Medicine. 1999;190(9):1329–41. 10544204 10.1084/jem.190.9.1329 PMC2195687

[57] Snapper SB, Rosen FS, Mizoguchi E, Cohen P, Khan W, Liu CH, et al Wiskott-Aldrich syndrome protein-deficient mice reveal a role for WASP in T but not B cell activation. Immunity. 1998;9(1):81–91. 9697838 10.1016/s1074-7613(00)80590-7

[58] Zhang JY, Shi FB, Badour K, Deng YP, McGavin MKH, Siminovitch KA. WASp verprolin homology, cofilin homology, and acidic region domain-mediated actin polymerization is required for T cell development. Proc Natl Acad Sci U S A. 2002;99(4):2240–5. 10.1073/pnas.042686099 11842211 PMC122349

[59] El Malki K, Karbach SH, Huppert J, Zayoud M, Reissig S, Schuler R, et al An alternative pathway of imiquimod-induced psoriasis-like skin inflammation in the absence of interleukin-17 receptor a signaling. J Invest Dermatol. 2013;133(2):441–51. 10.1038/jid.2012.318 22951726

[60] Kondo M, Weissman IL, Akashi K. Identification of clonogenic common lymphoid progenitors in mouse bone marrow. Cell. 1997;91(5):661–72. 9393859 10.1016/s0092-8674(00)80453-5

