# Supplementary figures and images for: The actin remodeling protein cofilin is crucial for thymic αβ but not γδ T-cell development
Source: PLoS Biol. 2018 Jul 9;16(7):e2005380. doi: 10.1371/journal.pbio.2005380 (PMC6053251; doi:10.1371/journal.pbio.2005380)

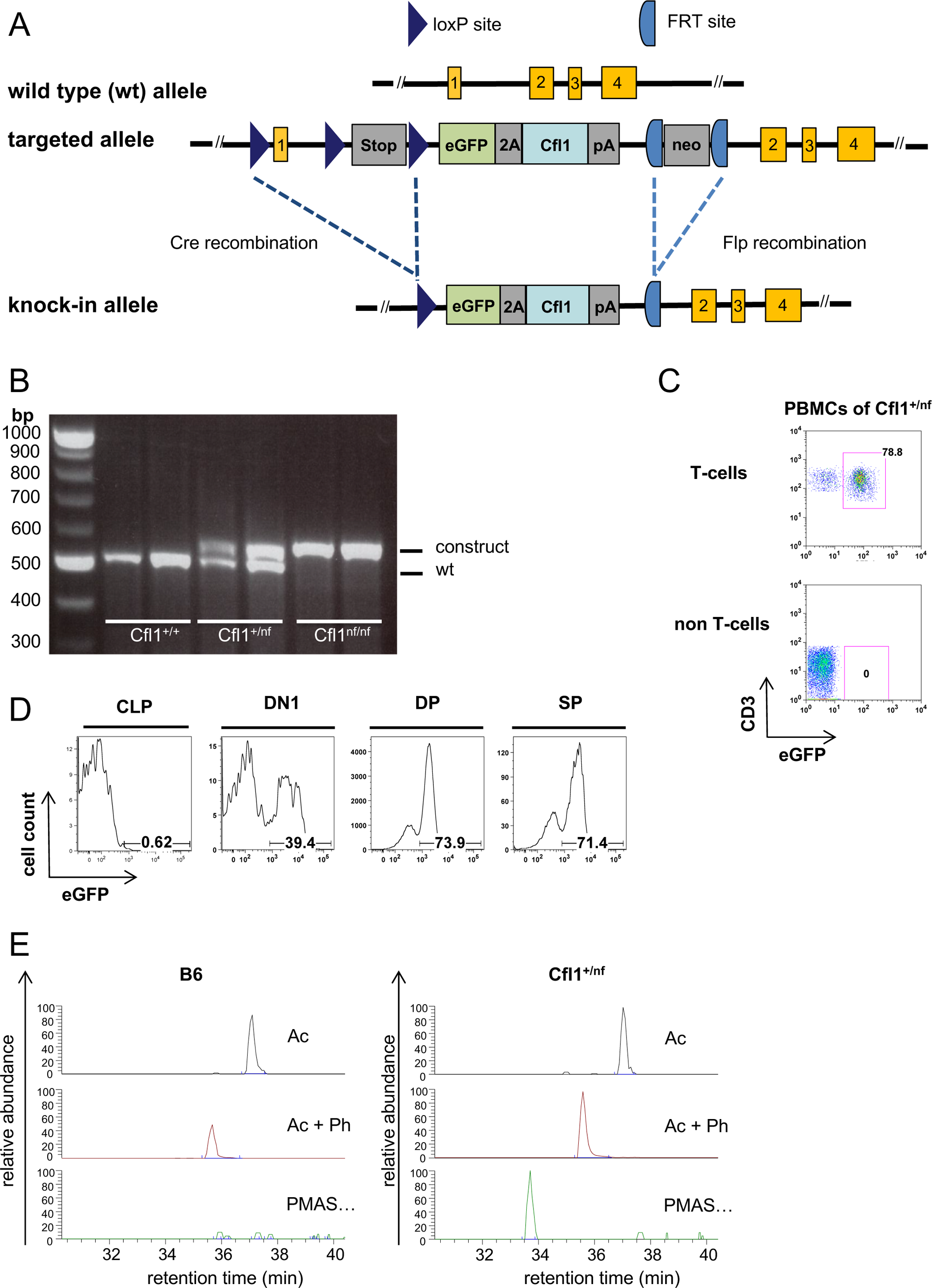

Supplement: S1 Fig — (A) Strategy used to create T-cell–specific nf cofilin knock-in mice. The first line shows the exon-intron organization of the mouse cofilin gene. It lies on chromosome 19 and consists of 4 exons (filled yellow boxes). In the targeted allele (second line) a floxed stop cassette, an eGFP-2A-Cfl1 sequence and a FRT-flanked neomycin (neo) cassette were inserted into the intronic region between exon 1 and 2 of the cofilin gene. Another loxP site was introduced into the noncoding sequence of exon 1. Mice carrying the construct in their germline were mated with Flp deleter mice in order to remove the neomycin cassette (third line). Afterwards, T-cell–specific knock-out of endogenous cofilin by deletion of exon 1 and at the same time knock-in of the eGFP-2A-Cfl1 expression cassette was achieved by crossing mice carrying the Flp recombined construct with Lck-Cre mice. (B) Mouse genotyping was performed by PCR of tail DNA. The allele carrying the construct could be discriminated from the WT allele by the additional loxP site. Cfl1+/+: wt mice; Cfl1+/nf: heterozygous mice; Cfl1nf/nf: homozygous mice. (C) Flow cytometric analysis of eGFP expression in T cells and non-T cells of purified peripheral blood mononuclear cells PBMCs from Cfl1+/nf mice. (D) Flow cytometric analysis of eGFP expression in common lymphoid progenitor cells CLPs from the bone marrow and thymocytes (DN1, DP and SP stage) from thymi of Cfl1+/nf mice. For analysis of eGFP expression in CLPs, lineage negative cells were isolated from BM of mice by MACS. CLPs were then identified by their expression of IL7Rα, c-kit and Sca-1 [60]. (E) LC-MS/MS analysis of cofilin peptides resulting from tryptic digestion of cofilin isolated from splenic T cells of B6 and Cfl1+/nf mice. Shown are the extracted ion chromatograms of the depicted peptides. “Ac” represents N-terminus of cofilin starts with acetylated alanine and serine is not phosphorylated; “Ac + Ph” represents N-terminus of cofilin starts with acetylated alanine [file pbio.2005380.s001.tif]

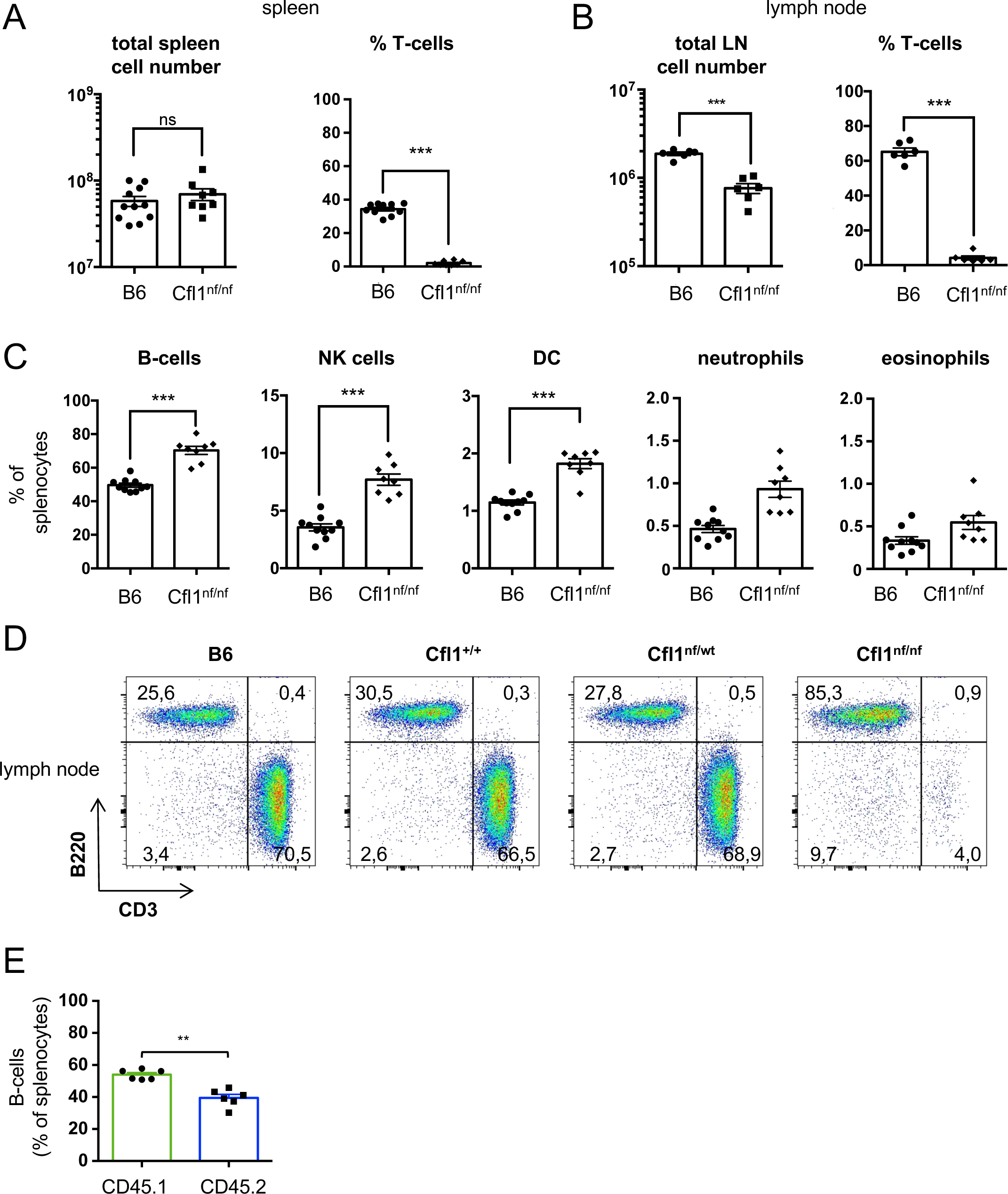

Supplement: S2 Fig — (A) Total spleen cell number and percentage of T cells in spleen of B6 mice and Cfl1nf/nf mice. (B) Total thymic cell number and percentage of T cells in LNs of B6 mice and Cfl1nf/nf mice. (C) Splenic cells were analyzed for B-cell, NK cell, DC, neutrophil, and eosinophil populations. Shown are the percentage of total splenocytes. Each data point represents an individual mouse. (D) Flow cytometric analysis of B- and T-cell populations in lymphocytes derived from LNs of control B6 mice, Cfl1+/+ mice (homozygous for construct but no Cre-mediated knock-in), Cfl1nf/wt (heterozygous for construct with Cre-mediated knock-in) and Cfl1nf/nf mice (homozygous for construct with Cre-mediated knock-in). One representative result out of 3 independent experiments with a total of 6 mice per group is shown. (E) Analysis of the percentage of splenic B-cells within the chimera (see Fig 2D) from both tester (CD45.2+) and competitor (CD45.1+) donor cells. Data is represented as mean ± SEM and summarizes 4 independent experiments with a total of ≥ 6 mice per group. **** p < 0.0001; ** p < 0.01; * p < 0.05. Underlying data can be found in S1 Data. ns, not significant. (TIF) [file pbio.2005380.s002.tif]

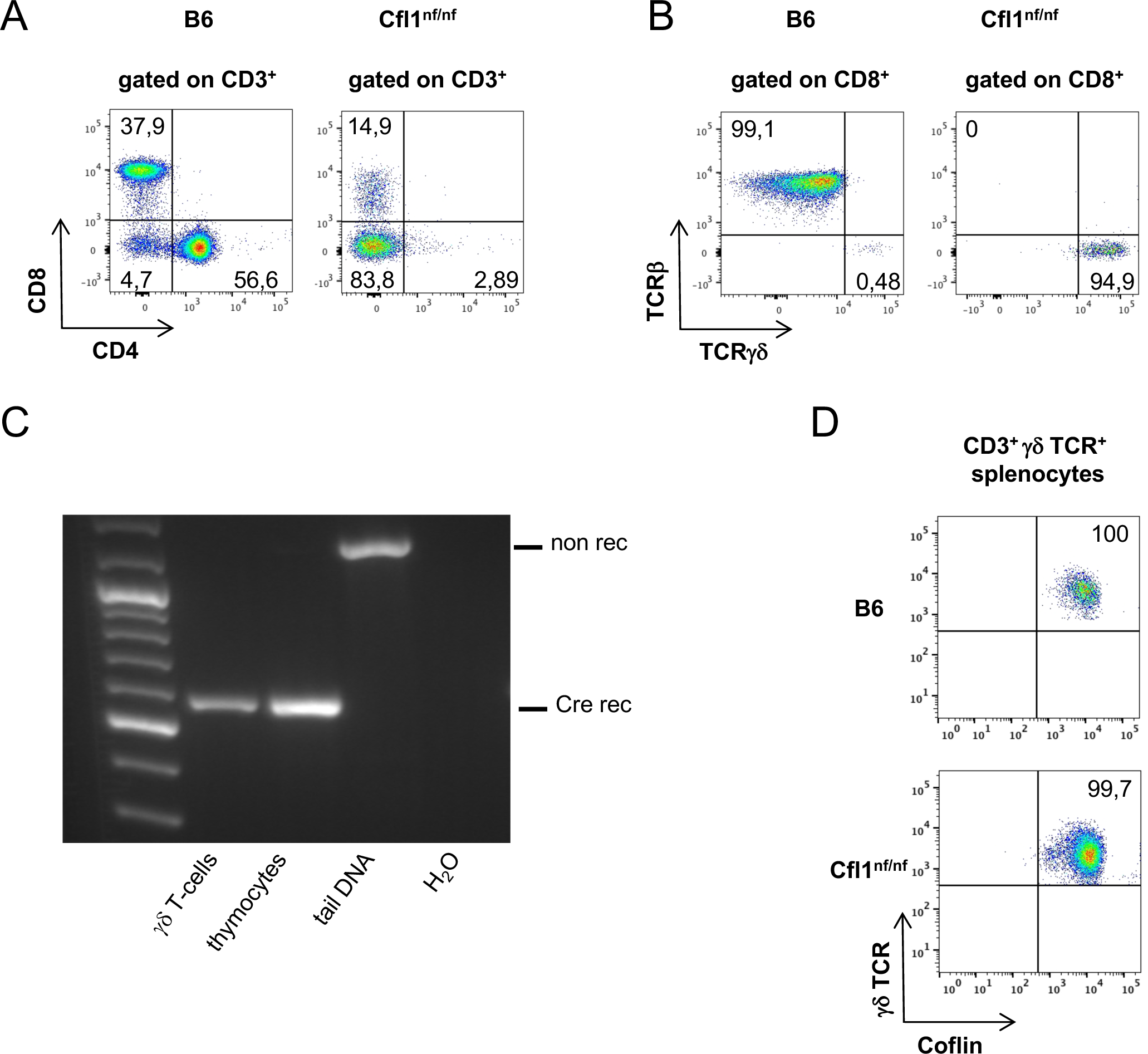

Supplement: S3 Fig — (A) Flow cytometric analysis of T-cell co-receptors CD4 and CD8 on splenic T cells of B6 and Cfl1nf/nf mice. (B) Flow cytometric analysis of T-cell populations in lymphocytes derived from spleen of control B6 mice (left panel) and Cfl1nf/nf mice (right panel). CD8+ T-cell population in spleen of B6 mice express either highly TCRβ or low amounts of TCRγδ. Splenic CD8+ T cells of Cfl1nf/nf mice express solely TCRγδ. (C) γδ T cells were isolated from splenocytes of Cfl1nf/nf mice via FACS sort and were analyzed for Cre recombination by PCR of cell lysates. Lysates of thymocytes were used as a positive control, whereas mouse tail DNA (from Cfl1nf/nf mice) and H2O served as negative controls. (D) Cofilin expression analysis of splenic γδ T cells of B6 mice (upper panel) and Cfl1nf/nf mice (lower panel). Cells were pre-gated on CD3+ γδ T cells. nf, nonfunctional. (TIF) [file pbio.2005380.s003.tif]

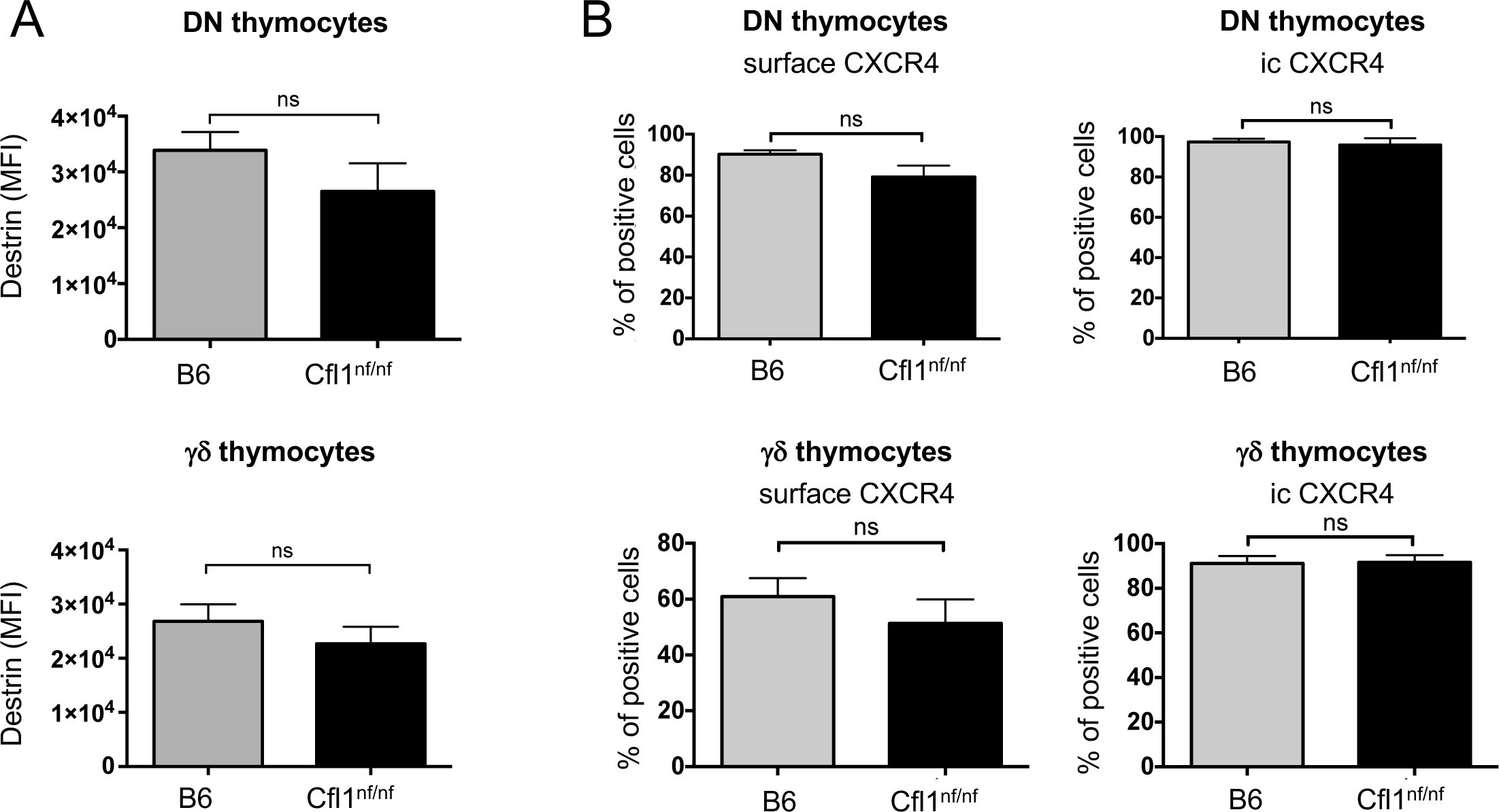

Supplement: S4 Fig — (A) Analysis of destrin expression in DN and γδ thymocytes of B6 and Cfl1nf/nf mice. (B) Analysis of ic and surface expression of CXCR4. Data is represented as mean ± SEM and summarizes 4 independent experiments with a total of ≥ 6 mice per group. **** p < 0.0001; ** p < 0.01; * p < 0.05. Underlying data can be found in S1 Data. ic, intracellular; ns, not significant. (TIF) [file pbio.2005380.s004.tif]
