# Supplementary material for: The actin remodeling protein cofilin is crucial for thymic αβ but not γδ T-cell development
Source: PLoS Biol. 2018 Jul 9;16(7):e2005380. doi: 10.1371/journal.pbio.2005380 (PMC6053251; doi:10.1371/journal.pbio.2005380)
Supplement: S1 Text — (DOCX) [file pbio.2005380.s005.docx]

**Supplemental Experimental Procedures**

### Mice

C57BL/6N mice (B6; CD45.2) were purchased from Janvier. Congenic B6-LY5.2/Cr (B6; CD45.1) mice were originally obtained from the Frederick National Laboratory for Cancer Research. B6.Tg(ROSA)26-FLP/Uhg (Flp deleter mice) were generously provided by Marc Freichel (University of Heidelberg) and B6.Tg(Lck-cre)548Jxm/ArndUhg (Lck-Cre) [1] were a kind gift of Jamey Marth (University of California, Santa Barbara).

### Generation of Cfl1^nf/nf^ mice

To generate Cfl1^nf/nf^ mice, a targeting vector was constructed containing the genomic sequence of the mouse cofilin gene (derived from BAC RPCIB731K05389Q, Source Bioscience) flanked by 1.1 kb (5’) and 5 kb (3’) homology arms. One loxP site was added into the non-coding region of exon 1. A floxed STOP cassette, followed by an eGFP-2A-Cfl1 expression cassette and a FRT-flanked neomycin selection cassette were inserted into the intron region between exon 1 and 2. Strain C57BL/6-derived JM8A1.N3 ES cells [2] were electroporated with linearized genomic DNA construct and selected for G418 resistance (performed at the Transgenic Core Facility of the German Cancer Research Center Heidelberg). Homologous recombination of the targeting construct was analyzed by Southern Blot. Positive clones were injected into mouse blastocysts to produce chimeric mice, which were further bred with Flp deleter mice to remove the neomycin cassette. Those offsprings which showed germline transmission of the targeting construct (B6-*Cfl1^tm1 (eGFP-2A-Cfl1)Uhg^*) and had lost the neomycin resistance gene were then mated with Lck-Cre mice to induce the T-cell specific knock-out of endogenous cofilin and at the same time knock-in of the eGFP-2A-cofilin expression cassette. Further intercrossing was performed to obtain homozygous mice (B6-*Cfl1^tm1 (eGFP-2A-Cfl1)Uhg^*Tg(Lck-cre)548Jxm/Uhg; short: Cfl1^nf/nf^).

Genotyping was done using primers amplifying the cofilin genomic region around exon 1 (where alleles carrying the construct can be discriminated from wt alleles by the additional loxP site) and by screening for the presence of the Cre gene.

### Immunoblotting

For preparation of samples, cells were lysed in reducing SDS-sample buffer by cooking at 95°C for 5 min. Samples were subjected to SDS-PAGE followed by immunoblotting. To determine the phosphorylation index of cofilin, membranes were probed for serine 3-phosphorylated cofilin (anti-phospho-cofilin Ser-3 antibody, Cell Signaling 3311L) followed by a staining with a cofilin antiserum (produced in our lab). As a loading control, an α-GAPDH antibody (Sigma-Aldrich) was used. Signals were quantified by densitometry.

### In-gel tryptic digestion and LC-MS/MS analysis

After SDS-PAGE coomassie stained bands were cut out with a scalpel and processed as described previously [3]. In brief, samples were reduced, alkylated and digested with trypsin. Peptides were extracted from the gel pieces, concentrated in a speedVac vacuum centrifuge and diluted to a total volume of 30 µl with 0.1% TFA. 25 µl of the sample was analyzed by a nanoHPLC system (nanoAcquity, Waters) coupled to an ESI LTQ Orbitrap mass spectrometer (Thermo Fisher). Sample was loaded on a C18 trapping column and separated on an analytical column (75 µm x 250 mm) with a flow rate of 300 nl/min in an acetonitrile-gradient (3%-40%). One survey scan (res: 60000) was followed by 5 information dependent product ion scans in the ion trap. The uninterpreted MS/MS spectra were searched against a small protein database (510 entries) containing wt and sequence variants of cofilin using the Mascot software (Matrix Science). Trypsin was specified as enzyme. Carbamidomethyl was set as fixed modification of cysteine and oxidation (methionine), deamidation (asparagines, glutamine), N-terminal acetylation and phosphorylation (serine, threonine) as variable modifications. Mass tolerance was set to 10 ppm and 0.5 Da for MS and MS/MS, respectively.

To detect unexpected modifications, error-tolerant searches were done. For comparison a synthetic peptide (PMASGVAVSDGVIKFNDMK, JPT, Berlin) was analyzed in the same way.

### Isolation of cell subtypes

For purification of DN thymocytes, CD4^-^ CD8^-^ thymocytes of B6 mice and Cfl1^nf/nf^ mice were sorted with BD FACSAriaIII. For γδ thymocyte analysis, DN thymocytes were pre-enriched by MACS negative selection employing magnetic beads against CD4 and CD8a (Miltenyi). Afterwards, to discriminate γδ thymocytes from non-γδ DN thymocytes, cells were stained for TCRγδ (GL3).

### Single cell preparation from skin and lung tissue

Ear tissue was digested in RPMI1640 + 10% FBS + 1 µM CaCl2 + 1 µM MgCl2 + 50 U/ mL DNAse I (Roche)+ 2 mg/mL Collagenase A (Roche) for 90 min at 37°C. Lung tissue was digested in RPMI1640 + 2 mg/mL Collagenase XI (Sigma Aldrich) for 90 min at 37°C.

Ear and lung tissue digestion was stopped by adding 10 mM EDTA for 10 min, before cell suspension was passed through 70 µm cell strainer. Single cell suspension was immediately stained for Vγ chain and γδ T cell surface markers and analyzed by flow cytometry.

### Flow cytometry

Monoclonal antibodies recognizing the following surface markers and molecules were used for flow cytometry: CD3 (145-2C11), CD4 (GK1.5), CD8 (53-6.7), CD11b (M1/70), CD11c (HL3), CD24 (M1/69), CD25 (PC61), CD27 (LG.7F9), CD44 (IM7), CD45.1 (A20), CD45.2 (104), CD45R-B220 (EA3-6B2), CD117 (2B8), CD122 (TM-β1), CD127 (A7R34), NK1.1 (PK136), Ly6A/E (D7), Ly6C (AL-21), Ly6G (1A8), TCRß (H57-597), TCRγδ (GL3), CXCR4 (L276F12), Destrin/ADF (GV-13). All were obtained from BD, eBioscience or BioLegend. 7AAD was used to discriminate dead cells. In order to stain intracellular molecules, cells that were already stained for surface markers were fixed with 1.5% PFA for 10 min at room temperature. Intracellular staining was performed in FACS buffer (PBS, 3% heat-inactivated FBS, 0.1% NaN_3_) containing 0.1% saponine. Cells were washed with the same buffer and resuspended in FACS buffer only for measurement. For staining of ex vivo restimulated lymph node cells of IMQ-treated mice, surface markers were stained for 10 min on ice before cells were fixed with 2% PFA and permeabilized with 1x eBioscience Permeabilization Buffer (Thermo Fischer Scientific) for 40 min on ice. Finally, cells were stained for intracellular cytokines and transcription factors (IL-17A, RORγt). All flow cytometric measurements were done on an LSRII (BD).

### Generation of mixed bone marrow chimeras

Bone marrow cells were isolated from tibia and femur of CD45.1^+^ B6 and CD45.2^+^ nf cofilin knock-in mice. CD3^+^ cells were removed with the help of an α-CD3 antibody (clone 500A2, BD) and Streptavidin-coupled Dynabeads M-280 according to manufacturer’s instructions. Tester (CD45.2^+^) and competitor cells (CD45.1^+^) were mixed in a 1:1 ratio and each 10 Mio of cells (in 300 µl PBS) were injected *i.v.* into lethally irradiated B6 mice. For γ-irradiation of recipient B6 mice, 1,100 Rad were administered in a split dose (2x 550 Rad; 3 h break apart) via a cesium isotope (^127^Cs) source the day before cell injection. These mice also received Amoxicillin (0.5 mg/ml; Sandoz) with their drinking water. Successful lymphocyte repopulation of recipient mice was determined via flow-cytometric staining of peripheral blood cells. Finally, mice were sacrificed 10 weeks after reconstitution and thymic populations were analyzed by flow cytometry.

**Supplemental References**

1. Orban PC, Chui D, Marth JD. Tissue- and site-specific DNA recombination in transgenic mice. Proceedings of the National Academy of Sciences of the United States of America. 1992;89(15):6861-5.

2. Pettitt SJ, Liang Q, Rairdan XY, Moran JL, Prosser HM, Beier DR, et al. Agouti C57BL/6N embryonic stem cells for mouse genetic resources. Nature methods. 2009;6(7):493-5.

3. Barenz F, Inoue D, Yokoyama H, Tegha-Dunghu J, Freiss S, Draeger S, et al. The centriolar satellite protein SSX2IP promotes centrosome maturation. The Journal of cell biology. 2013;202(1):81-95.
